# Supplementary material for: Early rehabilitation to prevent postintensive care syndrome in patients with critical illness: a systematic review and meta-analysis
Source: BMJ Open. 2018 May 5;8(5):e019998. doi: 10.1136/bmjopen-2017-019998 (PMC5942437; doi:10.1136/bmjopen-2017-019998)
Supplement: Supplementary data [file bmjopen-2017-019998supp001.pdf]

**Supplementary file 1.**

The full search strategies for Medline (via PubMed), EMBASE and the Cochrane Central Register of clinical trials.

#1 ("critical ill" or "critical illness" or "critical care" or "intensive care" or "mechanical ventilation" or "mechanical ventilated" or "postoperative care")

#2 (rehabilitation or "physical therapy" or physiotherapy or exercise or mobilization or "mobility intervention" or "muscle training")

#3 ("Activities of Daily Living" or "Quality of Life" or "post-intensive care syndrome" or "motor function" or "Physical Functioning" or "functional status" or "physical function" or "ventilator days" or "quality of life" or (walking or walk) or muscle or polyneuromyopathy' or "length of stay" or "length of ICU stay" or "length of hospital stay" or "intubation period" or "duration of mechanical ventilation" or re-admission or "functional outcome" or "ICU-acquired weakness" or "ICU-acquired paresis" or ICUAW or "ICU-AW" or "intensive care unit acquired weakness" or "critical illness polyneuropathy" or "critical illness myopathy" or "critical illness neuromyopathy" or "acute quadriplegic myopathy" or "thick filament myopathy" or "acute necrotizing myopathy of intensive care" or "acute corticosteroid myopathy" or "critical illness neuromuscula syndromes" or "Tower test" or "Timed Up and Go Test" or "dysexecutive questionnaire" or FAQ or "EQ-5D VAS" or 6MWD or "6-min walking distance" or "Quadriceps force, and self-perceived functional status" or "SF-36 PF" or MRC or "Medical Research Council" or "AQoL utility" or "EQ-5D" or PFIT or "physical functional ICU test" or "Hospital Anxiety and Depression Scale" or "Hand-grip strength")

#4 #1 AND #2 AND #3

Article search was performed at 7 July 2016 and a total of 5104 articles are scanned.
