# Supplementary material for: Early rehabilitation to prevent postintensive care syndrome in patients with critical illness: a systematic review and meta-analysis
Source: BMJ Open. 2018 May 5;8(5):e019998. doi: 10.1136/bmjopen-2017-019998 (PMC5942437; doi:10.1136/bmjopen-2017-019998)
Supplement: Supplementary data [file bmjopen-2017-019998supp002.pdf]

Supplementary file 2

List of excluded studies

40 excluded studies with reasons

10 No early rehabilitation [1-10]

10 No physical rehabilitation [11-20]

5 Systematic review and meta-analysis [21-25]

4 Initiated rehabilitation before ICU admission [26-29]

3 Protocol paper [30-32]

2 Not ICU patients [33 34]

1 Same timing rehabilitation initiated between two groups [35]

5 Others [36-40]

References

1. Stein R, Maia CP, Silveira AD, et al. Inspiratory muscle strength as a determinant of functional capacity early after coronary artery bypass graft surgery. *Archives of physical medicine and rehabilitation* 2009;90(10):1685-91. doi: 10.1016/j.apmr.2009.05.010
2. Patman S, Sanderson D, Blackmore M. Physiotherapy following cardiac surgery: is it necessary during the intubation period? *The Australian journal of physiotherapy* 2001;47(1):7-16.
3. Porta R, Vitacca M, Gile LS, et al. Supported arm training in patients recently weaned from mechanical ventilation. *Chest* 2005;128(4):2511-20. doi: 10.1378/chest.128.4.2511
4. Jackson JC, Ely EW, Morey MC, et al. Cognitive and physical rehabilitation of intensive care unit survivors: results of the RETURN randomized controlled pilot investigation. *Crit Care Med* 2012;40(4):1088-97. doi: 10.1097/CCM.0b013e3182373115
5. Patman S, Jenkins S, Stiller K. Physiotherapy does not prevent, or hasten recovery from, ventilator-associated pneumonia in patients with acquired brain injury. *Intensive Care Med* 2009;35(2):258-65. doi: 10.1007/s00134-008-1278-2
6. Chen S, Su CL, Wu YT, et al. Physical training is beneficial to functional status and survival in patients

with prolonged mechanical ventilation. *Journal of the Formosan Medical Association = Taiwan yi zhi* 2011;110(9):572-9. doi: 10.1016/j.jfma.2011.07.008

7. Walsh TS, Salisbury LG, Merriweather JL, et al. Increased Hospital-Based Physical Rehabilitation and Information Provision After Intensive Care Unit Discharge: The RECOVER Randomized Clinical Trial. *JAMA internal medicine* 2015;175(6):901-10. doi: 10.1001/jamainternmed.2015.0822
8. Chiang LL, Wang LY, Wu CP, et al. Effects of physical training on functional status in patients with prolonged mechanical ventilation. *Phys Ther* 2006;86(9):1271-81. doi: 10.2522/ptj.20050036
9. Chen YH, Lin HL, Hsiao HF, et al. Effects of exercise training on pulmonary mechanics and functional status in patients with prolonged mechanical ventilation. *Respir Care* 2012;57(5):727-34. doi: 10.4187/respcare.01341
10. Burtin C, Clerckx B, Robbeets C, et al. Early exercise in critically ill patients enhances short-term functional recovery. *Crit Care Med* 2009;37(9):2499-505. doi: 10.1097/CCM.0b013e3181a38937
11. Jackson JC, Girard TD, Gordon SM, et al. Long-term cognitive and psychological outcomes in the awakening and breathing controlled trial. *Am J Respir Crit Care Med* 2010;182(2):183-91. doi: 10.1164/rccm.200903-0442OC
12. Nava S. Rehabilitation of patients admitted to a respiratory intensive care unit. *Archives of physical medicine and rehabilitation* 1998;79(7):849-54.
13. Mackay MR, Ellis E, Johnston C. Randomised clinical trial of physiotherapy after open abdominal surgery in high risk patients. *The Australian journal of physiotherapy* 2005;51(3):151-9.
14. Stiller K, Montarello J, Wallace M, et al. Efficacy of breathing and coughing exercises in the prevention of pulmonary complications after coronary artery surgery. *Chest* 1994;105(3):741-7.
15. Caruso P, Denari SD, Ruiz SA, et al. Inspiratory muscle training is ineffective in mechanically ventilated critically ill patients. *Clinics (Sao Paulo)* 2005;60(6):479-84. doi: /S1807-59322005000600009
16. Albert NM, Gillinov AM, Lytle BW, et al. A randomized trial of massage therapy after heart surgery. *Heart Lung* 2009;38(6):480-90. doi: 10.1016/j.hrtlng.2009.03.001
17. Pattanshetty RB, Gaude GS. Effect of multimodality chest physiotherapy on the rate of recovery and prevention of complications in patients with mechanical ventilation: a prospective study in medical and surgical intensive care units. *Indian J Med Sci* 2011;65(5):175-85. doi: 10.4103/0019-5359.106608
18. Templeton M, Palazzo MG. Chest physiotherapy prolongs duration of ventilation in the critically ill ventilated for more than 48 hours. *Intensive Care Med* 2007;33(11):1938-45. doi: 10.1007/s00134-007-0762-4
19. Cader SA, Vale RG, Castro JC, et al. Inspiratory muscle training improves maximal inspiratory pressure and may assist weaning in older intubated patients: a randomised trial. *Journal of physiotherapy* 2010;56(3):171-7.

20. Pattanshetty RB, Gaude GS. Effect of multimodality chest physiotherapy in prevention of ventilator-associated pneumonia: A randomized clinical trial. *Indian journal of critical care medicine : peer-reviewed, official publication of Indian Society of Critical Care Medicine* 2010;14(2):70-6. doi: 10.4103/0972-5229.68218
21. Patel BK, Pohlman AS, Hall JB, et al. Impact of early mobilization on glycemic control and ICU-acquired weakness in critically ill patients who are mechanically ventilated. *Chest* 2014;146(3):583-9. doi: 10.1378/chest.13-2046
22. Connolly B, Salisbury L, O'Neill B, et al. Exercise rehabilitation following intensive care unit discharge for recovery from critical illness. *Cochrane Database Syst Rev* 2015(6):CD008632. doi: 10.1002/14651858.CD008632.pub2
23. Mehrholz J, Pohl M, Kugler J, et al. Physical rehabilitation for critical illness myopathy and neuropathy. *Cochrane Database Syst Rev* 2015(3):CD010942. doi: 10.1002/14651858.CD010942.pub2
24. Elkins M, Dentice R. Inspiratory muscle training facilitates weaning from mechanical ventilation among patients in the intensive care unit: a systematic review. *Journal of physiotherapy* 2015;61(3):125-34. doi: 10.1016/j.jphys.2015.05.016
25. Castro-Avila AC, Seron P, Fan E, et al. Effect of Early Rehabilitation during Intensive Care Unit Stay on Functional Status: Systematic Review and Meta-Analysis. *PloS one* 2015;10(7):e0130722. doi: 10.1371/journal.pone.0130722
26. Herdy AH, Marcchi PL, Vila A, et al. Pre- and postoperative cardiopulmonary rehabilitation in hospitalized patients undergoing coronary artery bypass surgery: a randomized controlled trial. *Am J Phys Med Rehabil* 2008;87(9):714-9. doi: 10.1097/PHM.0b013e3181839152
27. Arthur HM, Daniels C, McKelvie R, et al. Effect of a preoperative intervention on preoperative and postoperative outcomes in low-risk patients awaiting elective coronary artery bypass graft surgery. A randomized, controlled trial. *Annals of internal medicine* 2000;133(4):253-62.
28. Fagevik Olsen M, Hahn I, Nordgren S, et al. Randomized controlled trial of prophylactic chest physiotherapy in major abdominal surgery. *Br J Surg* 1997;84(11):1535-8.
29. Weiner P, Zeidan F, Zamir D, et al. Prophylactic inspiratory muscle training in patients undergoing coronary artery bypass graft. *World journal of surgery* 1998;22(5):427-31.
30. Kayambu G, Boots RJ, Paratz JD. Early rehabilitation in sepsis: a prospective randomised controlled trial investigating functional and physiological outcomes The i-PERFORM Trial (Protocol Article). *BMC anesthesiology* 2011;11:21. doi: 10.1186/1471-2253-11-21
31. Thomas K, Wright SE, Watson G, et al. Extra Physiotherapy in Critical Care (EPICC) Trial Protocol: a randomised controlled trial of intensive versus standard physical rehabilitation therapy in the critically ill. *BMJ open* 2015;5(5):e008035. doi: 10.1136/bmjopen-2015-008035
32. Brummel NE, Jackson JC, Girard TD, et al. A combined early cognitive and physical rehabilitation

program for people who are critically ill: the activity and cognitive therapy in the intensive care unit (ACT-ICU) trial. *Phys Ther* 2012;92(12):1580-92. doi: 10.2522/ptj.20110414

33. Reeve JC, Nicol K, Stiller K, et al. Does physiotherapy reduce the incidence of postoperative pulmonary complications following pulmonary resection via open thoracotomy? A preliminary randomised single-blind clinical trial. *Eur J Cardiothorac Surg* 2010;37(5):1158-66. doi: 10.1016/j.ejcts.2009.12.011
34. Lee SM, Kang SB, Jang JH, et al. Early rehabilitation versus conventional care after laparoscopic rectal surgery: a prospective, randomized, controlled trial. *Surgical endoscopy* 2013;27(10):3902-9. doi: 10.1007/s00464-013-3006-4
35. Hirschhorn AD, Richards DA, Mungovan SF, et al. Does the mode of exercise influence recovery of functional capacity in the early postoperative period after coronary artery bypass graft surgery? A randomized controlled trial. *Interactive cardiovascular and thoracic surgery* 2012;15(6):995-1003. doi: 10.1093/icvts/ivs403
36. Yosef-Brauner O, Adi N, Ben Shahar T, et al. Effect of physical therapy on muscle strength, respiratory muscles and functional parameters in patients with intensive care unit-acquired weakness. *The clinical respiratory journal* 2015;9(1):1-6. doi: 10.1111/crj.12091
37. Denehy L, Skinner EH, Edbrooke L, et al. Exercise rehabilitation for patients with critical illness: a randomized controlled trial with 12 months of follow-up. *Crit Care* 2013;17(4):R156. doi: 10.1186/cc12835
38. Melchers P, Maluck A, Suhr L, et al. An early onset rehabilitation program for children and adolescents after traumatic brain injury (TBI): Methods and first results. *Restorative neurology and neuroscience* 1999;14(2):153-60.
39. Dantas CM, Silva PF, Siqueira FH, et al. Influence of early mobilization on respiratory and peripheral muscle strength in critically ill patients. *Revista brasileira de terapia intensiva* 2012;24(2):173-8.
40. Dong ZH, Yu BX, Sun YB, et al. Effects of early rehabilitation therapy on patients with mechanical ventilation. *World journal of emergency medicine* 2014;5(1):48-52. doi: 10.5847/wjem.j.1920-8642.2014.01.008
