# Supplementary material for: Early rehabilitation to prevent postintensive care syndrome in patients with critical illness: a systematic review and meta-analysis
Source: BMJ Open. 2018 May 5;8(5):e019998. doi: 10.1136/bmjopen-2017-019998 (PMC5942437; doi:10.1136/bmjopen-2017-019998)
Supplement: Author's manuscript [file bmjopen-2017-019998.draft_revisions.pdf]

BMJ Open

BMJ Open is committed to open peer review. As part of this commitment we make the peer review history of every article we publish publicly available.

When an article is published we post the peer reviewers' comments and the authors' responses online. We also post the versions of the paper that were used during peer review. These are the versions that the peer review comments apply to.

The versions of the paper that follow are the versions that were submitted during the peer review process. They are not the versions of record or the final published versions. They should not be cited or distributed as the published version of this manuscript.

BMJ Open is an open access journal and the full, final, typeset and author-corrected version of record of the manuscript is available on our site with no access controls, subscription charges or pay-per-view fees (http://bmjopen.bmj.com).

If you have any questions on BMJ Open's open peer review process please email info.bmjopen@bmj.com

BMJ Open

Early rehabilitation to prevent post-intensive care syndrome in critically ill patients: a systematic review and meta-analysis

Journal:	*BMJ Open*
Manuscript ID	bmjopen-2017-019998
Article Type:	Research
Date Submitted by the Author:	19-Oct-2017
Complete List of Authors:	Fuke, Ryota; Tohoku Ika Yakka Daigaku Wakabayashi Byoin<br>Hifumi, Toru; Kagawa University Hospital, Emergency Medical Center<br>Kondo, Yutaka; Ryukyu Daigaku,<br>Hatakeyama, Junji; Yokohama Shiritsu Minato Sekijuji Byoin<br>Takei, Tetsuhiro; Yokohama Shiritsu Minato Sekijuji Byoin, Intensive Care Unit<br>Yamakawa, Kazuma; Osaka General Medical Center, Department of Emergency and Critical Care<br>Inoue, Shigeaki; Tokai University Hachioji city Hospital, Department of Emergency and Critical Care Medicine<br>Nishida, Osamu; Fujita Hoken Eisei Daigaku Igakubu Daigakuin Igaku Kenkyuka
Keywords:	early rehabilitation, intensive care unit, post-intensive care syndrome, sepsis

SCHOLARONE™
Manuscripts

Early rehabilitation to prevent post-intensive care syndrome in critically ill patients: a systematic review and meta-analysis

Ryota Fuke, MD[1*], Toru Hifumi, MD[2*], Yutaka Kondo, MD, PhD[3], Junji Hatakeyama, MD[4], Tetsuhiro Takei, MD, PhD[4], Kazuma Yamakawa, MD, PhD[5], Shigeaki Inoue, MD, PhD, MSCI[6], Osamu Nishida, MD, PhD[7]

[1] Division of Infectious Diseases and Infection Control, Tohoku Medical and Pharmaceutical University, Sendai City, Miyagi, JAPAN

[2] Emergency Medical Center, Kagawa University Hospital, Kita-gun-Takamatsu, JAPAN

[3] Department of Emergency and Critical Care Medicine, Graduate School of Medicine, University of the Ryukyus, Nakagami-gun, Okinawa, JAPAN

[4] Department of Intensive Care Medicine, Yokohama City Minato Red Cross Hospital, Yokohama City, Kanagawa, JAPAN

[5] Division of Trauma and Surgical Critical Care, Osaka General Medical Center, Osaka City, Osaka, JAPAN

[6] Department of Emergency and Critical Care Medicine, Tokai University Hachioji Hospital, Hachioji, Tokyo, JAPAN

[7] Department of Anaesthesiology and Critical Care Medicine, Fujita Health University School of Medicine, Toyoake City, Aichi, JAPAN

* Ryota Fuke and Toru Hifumi contributed equally to this manuscript.

Address correspondence and reprint requests to: Shigeaki Inoue, Department of Emergency and Critical Care Medicine, Tokai University Hachioji Hospital, Ishikawa-cho 1838, Hachioji City,

For peer review only

For peer review only - http://bmjopen.bmj.com/site/about/guidelines.xhtml

Tokyo 192-0032, Japan. Tel.: +81-42-639-1111; E-mail caf55000@gmail.com

Running title: Early rehabilitation to prevent post-intensive care syndrome

Keywords: early rehabilitation, intensive care unit, post-intensive care syndrome, sepsis

Conflict of interest: The authors declare that they have no conflict of interest.

Word count: 2939 words

Number of tables: 2

Number of figures: 4

Number of supplementary files for online-only publication: 2

Number of references: 44

For peer review only

For peer review only - http://bmjopen.bmj.com/site/about/guidelines.xhtml

ABSTRACT

Introduction: We examined the effectiveness of early rehabilitation for the prevention of post-intensive care syndrome (PICS), characterized as impairments in physical, cognitive, or mental health status in survivors of critical illness.**Methods**: We performed a systematic literature search in databases (Medline, Embase, and Cochrane Central Register of Controlled Trials) as well as a manual search to identify randomized controlled trials (RCTs) comparing the effectiveness of early rehabilitation versus standard care or no early rehabilitation for the prevention of PICS. **The primary outcomes were short-term physical-, cognitive-, and mental health-related outcomes, accessed during hospitalization. The secondary outcomes were long-term health-related QOL scores (standardized health-related quality of life with EuroQol 5 Dimension [EQ5D] and the Medical Outcomes Study 36-Item Short Form Health Survey Physical Function scale [SF-36 PF]).** We used the Grading of Recommendations Assessment, Development and Evaluation (GRADE) approach to rate quality of evidence (QoE). **Results**: From 5,105 screened abstracts, **6** RCTs were included. Early rehabilitation significantly improved short-term outcomes regarding Physical-related outcomes, which included an increased Medical Research Council scale score (MRC score) [standardized mean difference (SMD) 0.38, 95% confidence interval (CI) 0.10 to 0.66, p = 0.009] (QoE: low), with a decreased incidence of ICU-acquired weakness (ICU-AW) [odds ratio (OR) 0.42, 95% CI 0.22 to 0.82, p = 0.01] (QoE: low), compared to standard care or no early rehabilitation. However, there was no difference between the two groups in cognitive-related delirium-free days (SMD -0.02, 95% CI -0.23 to 0.20) (QoE: low) and mental health-related Hospital Anxiety and Depression Scale score (OR 0.79, 95% CI 0.29 to 2.12) (QoE: low). Early rehabilitation did not improve long-term outcomes of PICS characterized by EQ5D and SF-36PF. **Conclusions**: Early rehabilitation improved **only short-term physical-related outcomes** in critically ill patients. Further large RCTs are needed.

For peer review only - http://bmjopen.bmj.com/site/about/guidelines.xhtml

Strengths and limitations of this study

- ✓ This meta-analysis revealed that early rehabilitation has a limited effect on the prevention of PICS, with a significant improvement of short-term physical-related outcomes, but not cognitive and mental status-related outcomes in critically ill patients.
- ✓ The main limitations of this meta-analysis are: (1) the small number of patients in the included RCTs; (2) the lack of detailed analysis of adverse effects and PICS domains after hospital discharge; and (3) no confirmation of the exact first day of initiation of rehabilitation in 3 of the 8 included RCTs.
- ✓ This meta-analysis is useful to clarify the effectiveness and limitation of early rehabilitation for the prevention of PICS in survivors of critical illness.

For peer review only

INTRODUCTION

Dramatic developments and improvements of technique, instruments, and education systems in intensive care units (ICUs) have reduced the mortality of critically ill patients in the past four decades [1]. However, this evolution of life-saving interventions has resulted in increasing numbers of critically ill patient survivors with impaired physical and mental ability to return to usual life [2]. Persistent physical, cognitive and mental impairments in surviving ICU patients are serious problems that prevent them from being discharged home and, once home, from returning to usual daily life [2 3].

Post-intensive care syndrome (PICS) was established as a new syndrome that describes new or worsening impairments in physical, cognitive, or mental health status arising after critical illness and persisting beyond acute care hospitalization to initiate improvements for ICU survivors, and their families, across the continuum of care [4]. Since the establishment of PICS in 2010, observational studies evaluating the independent factors associated with PICS have been conducted [5]; however, few intervention studies targeting the prevention of PICS are available.

Physiotherapy with early rehabilitation is seen as an integral component of the multidisciplinary management of patients in ICUs. Exercise in other populations has been shown to improve strength and function, decrease inflammation [6-8], and affect oxidative stress [9-12]; hence, it has been suggested that early physiotherapy for ICU patients may prevent or reverse some physical impairment. However, there is no systematic review investigating the effectiveness of early rehabilitation for the prevention of PICS in ICU patients. The purpose of this systematic review was to assess the effectiveness of early rehabilitative interventions for the prevention of PICS in ICU patients.

For peer review only - http://bmjopen.bmj.com/site/about/guidelines.xhtml

METHODS

Protocols and registration

This review protocol has been registered in PROSPERO, an International Prospective Register of Systematic Reviews at the National Institute for Health Research and Center for Reviews and Dissemination (CRD) at the University of York (http://www.crd.york.ac.uk/PROSPERO/) (Registration No. CRD42016039759) [13]. This protocol followed the Preferred Reporting Items for Systematic Reviews and Meta-Analyses Protocols (PRISMA) statements [14 15], and the systematic review was reported following PRISMA guidelines [14 16 17]. The protocol for this systematic review has been published [18].

Search strategy

We searched Medline (via PubMed, 1996 to 7 June 2016), Embase (until 7 June 2016), and the Cochrane Central Register of Controlled Trials (CENTRAL) (until 7 June 2016). We were conducted to retrieve relevant articles for the literature review and searched full-text clinical trials conducted in humans. We also performed hand-searching to retrieve all potentially relevant articles from 8 June to 31 August. Key search terms to identify potential studies are listed in Supplementary file 1.

Study selection and inclusion criteria

The authors (YK, RF, SI) performed the first line comprehensive literature search and filter for duplicates. After duplicates were removed, two randomly chosen authors independently screened study titles and abstracts for potential relevance. **The kappa value for selection bias was 0.396.** When disagreement was identified between reviewers, the full text of the paper was retrieved; disagreements were again considered and discussed until consensus was reached. The full text of

For peer review only - http://bmjopen.bmj.com/site/about/guidelines.xhtml

articles included in the final selection was reviewed by two randomly chosen authors. We included trials with the following characteristics:

Study types: Randomized controlled trials (RCTs) were included, and non-randomized and observational studies were excluded.

Population: Adult (aged over 18 years) patients admitted to the ICU. We excluded patients with traumatic brain injury and stroke who did not fulfill PICS criteria; namely, those with acquired physical and psychiatric/cognitive dysfunction after ICU admission.

Intervention: The intervention was early rehabilitation. "Early" was defined as (1) starting at an earlier point than usual care or (2) being conducted within 7 days of ICU admission. "Rehabilitation" included all physiotherapy, occupational therapy, and palliative care related support. We excluded RCTs in which rehabilitation was initiated before ICU admission and RCTs comparing early rehabilitation with another intervention.

Control: Control group underwent standard care or no early rehabilitation.

Outcome: **The primary outcomes (short-term outcomes assessed during hospitalization) were as follows:** (1) physical-related outcomes(incidence of ICU-AW, the Medical Research Council (MRC) [19] scale score), (2) cognitive-related outcomes, using delirium-free days; and (3) mental status-related outcomes using the Hospital Anxiety and Depression Scale (HADS)[20]. **The secondary outcomes (long-term outcomes assessed post-discharge)** were standardized Health Related Quality of Life with EuroQol 5 Dimensions (EQ5D) [21 22], the Medical Outcomes Study 36-Item Short Form Health Survey Physical Function scale (SF-36 PF) [23] score as long-term physical function score.

Assessment of risk of bias

To assess the quality of included studies, we adopted the Cochrane risk of bias tool [24]. Randomly

For peer review only - http://bmjopen.bmj.com/site/about/guidelines.xhtml

chosen- two authors performed the risk of bias assessment, with disagreements resolved by discussion, and by third reviewer opinion if necessary. We considered the risk of bias for each element to be 'high' when bias is present and likely to affect outcomes, and 'low' when bias is not present, or present but unlikely to affect outcomes [25].

Rating the quality of evidence (QoE) using the GRADE approach

Two authors (RF and TH) independently used the Grading of Recommendations Assessment, Development and Evaluation (GRADE) tool to rate the quality of evidence (QoE). We applied the GRADE approach to rate QoE of early rehabilitation for outcomes. Although QoE represents a continuum, we assessed the quality of the body of evidence for each outcome categorized as high, moderate, low, or very low using the GRADE pro Guideline Development Tool.

Data extraction and management

Author(s), title, journal name, year of publication, website URL, and abstract were identified. Conference abstracts were excluded. Data from each study were extracted independently by two review authors. When faced with insufficient or incomplete data, we contacted the authors of the studies directly

Information related to the study population, sample size, interventions, comparators, potential biases in the conduct of the trial, outcomes including adverse events, follow-up, and methods of statistical analysis were extracted from the original reports into specially designed paper forms and then entered onto a spreadsheet.

Summarizing data and treatment effect

We performed a meta-analysis because data was available in several trials according to the

For peer review only - http://bmjopen.bmj.com/site/about/guidelines.xhtml

"Cochrane Handbook for Systematic Reviews of Interventions" and PRISMA guidelines by using Review Manager software (RevMan 5.3, Copenhagen, Denmark: The Nordic Cochrane Centre, the Cochrane Collaboration 2014). We summarized the results of the meta-analysis using the generic inverse variance method to facilitate pooling of estimates of treatment effect. We used odds ratios (ORs) with 95% confidence intervals (CIs) for dichotomous outcomes, and mean differences or standardized mean differences with 95% CI for continuous outcomes, when appropriate. If quantitative synthesis was not appropriate for a particular outcome, we made a plan providing a qualitative summary for that outcome.

Assessment of heterogeneity

We assessed for heterogeneity between trials for each outcome using I^2 statistics for quantifying inconsistency. We considered that significant heterogeneity was present when the reason for heterogeneity could not be explained and I^2 was 50% or greater. The presence of strong clinical heterogeneity was considered in the decision to conduct quantitative synthesis of data or to perform sensitivity analyses with a special focus [26].

Assessment of reporting bias

We investigated the possibility of publication bias using a funnel plot. To test for funnel plot asymmetry, we used the Egger test using STATA SE Statistical Software (Release 13. College Station, TX: StataCorp LP) [27 28].

Data synthesis

Estimates were pooled using a random effects model. We performed our analysis based on all published data or data made available to us [25].

For peer review only - http://bmjopen.bmj.com/site/about/guidelines.xhtml

RESULTS

Literature search

The PRISMA flow chart of study selection is shown in Figure 1. The combined search strategy identified 5105 citations, of which **46** were judged to be potentially eligible based on the abstract, and their full texts were obtained. After excluding **40** articles following full-text review (Supplementary file 2), 6 RCTs enrolling 709 patients were judged to be eligible and were included in the review [29-34].

Characteristics of included studies

Study characteristics are summarized in Table 1. Early rehabilitation was initiated within 3 days (median or mean) of ICU admission in 5 studies [29 31-34]. The first day of rehabilitation was not confirmed in 3 studies, although "early rehabilitation" was described in the text and we have contacted the authors to acquire additional information [30]. Rehabilitation strategies received in the intervention group varied in each protocol.

Risk of bias in the included trials

The risk of bias evaluated in each included RCT is shown in the risk of bias summary for PICS-related outcomes without in Figure 2. Regarding blinding, 8 trials were open-label RCTs. In 42 RCTs, blinding of outcome assessment was not described. In all RCTs, attrition bias was observed due to per-protocol analysis, inclusion of many patients who dropped out of the study, and death. Regarding mortality as an outcome measure, performance bias was low, because blinding did not affect mortality.

For peer review only - http://bmjopen.bmj.com/site/about/guidelines.xhtml

Outcomes

Quality assessment and a summary of each outcome measure are shown in Table 2.

<u>Primary outcomes (Short-term outcomes)</u>

(i) Physical-related outcomes

Incidence of ICU-AW

Two RCTs reported the incidence of ICU-AW (Figure 3). There was a significant reduction in the incidence of ICU-AW in the early rehabilitation group according to the random effects model (2 trials, n = 154; OR 0.42, 95% CI 0.22 to 0.82, p = 0.01; I^2 = 0%).

Risk of bias was determined to be serious because all RCTs included a moderate risk of bias. Imprecision was also determined to be serious due to the small number of included patients as per optimal information size (OIS) criteria. Thus, QoE was downgraded from 'high' to 'low' due to the serious risk of bias and imprecision.

MRC

Three RCTs reported MRC scores (Figure 3). We found significant improvement in the early rehabilitation group [MRC (3 trials, n = 196; ln(OR) 0.38, 95% CI 0.10 to 0.66, p = 0.009; I^2 = 0%)].

(ii) Cognitive-related outcomes

Delirium-free days

Two RCTs reported the incidence of delirium-free days (Figure 3). There was no significant increase in the delirium-free days in the early rehabilitation group according to a random effects model (2 trials, n = 326; ln(OR) -0.02, 95% CI -0.23 to 0.20, p = 0.62; I^2 = 0%). Risk of bias was determined

For peer review only - http://bmjopen.bmj.com/site/about/guidelines.xhtml

to be serious because all RCTs included moderate bias risk, and imprecision was also determined to be serious owing to the small number of included patients as per OIS criteria. Thus, QoE was downgraded from "high" to "low" owing to the serious risk of bias and imprecision.

(iii) Mental health-related outcomes

Incidence of HAS/HADS

Two RCTs reported incidence of HAS/HADS (Figure 3). There was no significant difference between the two groups according to a random effects model (2 trials, n = 92; OR 0.79, 95% CI 0.29 to 2.12, p = 0.64; I^2 = 0%). Risk of bias was determined to be serious because all RCTs included a moderate bias risk, and imprecision was also determined to be serious owing to the small number of included patients. Thus, QoE was downgraded from "high" to "low" owing to the serious risk of bias and imprecision.

Secondary outcomes (Long-term outcomes)

EQ5D

Two RCTs reported EQ5D (Figure 4). There was no significant difference between two groups according to a random effects model (2 trials, n = 63; ln(OR) 0.11, 95% CI -0.86 to 1.09, p = 0.82; I^2 = 72%). Risk of bias was judged serious because all RCTs demonstrated moderate bias risk, and inconsistency was judged serious due to severe heterogeneity (p = 0.06, I^2 = 72%). Imprecision was also judged serious due to the small number of included patients. Thus, QoE was downgraded from 'high' to 'very low' due to serious risk of bias, inconsistency, and imprecision.

SF-36 PF

Two RCTs reported SF-36 PF scale score (Figure 4). SF-36 PF was evaluated several months later.

For peer review only - http://bmjopen.bmj.com/site/about/guidelines.xhtml

Moreover, the timing of evaluation of SF-36 PF varied greatly i.e., at hospital discharge and at 2, 4, and 6 months after enrollment [34], or at 6 months after hospital discharge [31]. We found significant improvement in the early rehabilitation group [SF-36 PF (2 trials, n = 191; ln(OR) 2.41, 95% CI -0.75 to 5.58, p = 0.14; $I^2 = 98\%$)]. SF-36 PF scale score was related to significant heterogeneity because of the different timings of SF-36 PF evaluation in each study. Imprecision was also judged to be serious due to the small number of included patients as per OIS criteria. Thus, QoE was downgraded from 'high' to 'very low' due to serious risk of bias and imprecision.

For peer review only - http://bmjopen.bmj.com/site/about/guidelines.xhtml

DISCUSSION

Summary of the main results

This meta-analysis revealed that early rehabilitation significantly improved short-term physical function assessed by the MRC scoring system and the incidence of ICU-AW in critically ill patients. However, it did not significantly improve cognitive and mental status-related outcomes or . These results suggest that early rehabilitation has a limited effect on the prevention of PICS in critically ill patients, which only reflects the improvement of short-term physical function.

Strengths of the review

To our knowledge, the present study is the first meta-analysis of comprehensive PICS based on the RCTs whose study intervention population was limited to early rehabilitation. Additionally, we used the GRADE approach in the review process, which can make judgments about quality of evidence, and strength of recommendations in a systematic and transparent manner. Thus, the present meta-analysis can determine whether critical care physicians should initiate early rehabilitation for critically ill patients, and provide new evidence on the effectiveness of physiotherapy interventions aimed at early mobilization [35].

Agreements and disagreements with other studies or reviews

There are four systematic reviews regarding the effect of rehabilitation [35-37] or early rehabilitation [38] during ICU stay on functional status.

Kayambu et al. reported in their meta-analysis that physical therapy in the ICU may improve muscle strength, physical function, quality of life, ventilator-free days, ICU stay, and hospital stay; however, they included one RCT with patients with laparotomy out of 10 RCTs, and one study

For peer review only

For peer review only - http://bmjopen.bmj.com/site/about/guidelines.xhtml

selected in this study initiated physical therapy 30 days after bed rest [36]. Therefore, the results cannot be directly applied in our clinical practice due to the heterogeneity of the study population. In the present study, early rehabilitation did not improve quality of life. The inconsistency of these results may result from different methods for assessing quality of life: the Medical Outcomes Study 36-Item Short Form survey score was used in Kayambu et al.' s study, whereas the EQ5D was used in the present study.

Castro-Avila et al. conducted a systematic review and meta-analysis regarding the effect of early rehabilitation during ICU stay on functional status [36], and the study reported by Denehy et al. was included [39]. However, this study was not included in our systematic review owing to implementation of early active rehabilitation in the control group. This was considered the main reason why they concluded that early rehabilitation was not associated with improvement of walking distance and incidence of ICU-acquired weakness.

Tipping et al. reported a systematic review regarding the effects of active mobilization and rehabilitation in the ICU on mortality and function [37], and the study reported by Moss et al. was included [40]. However, because rehabilitation was not initiated in the early phase, this study was also not included in the current systematic review.

Clinical implementation

Initiation of early rehabilitation improved short-term outcome, such as the physical function score and the incidence of ICU-AW in the present study; however, it did not improve. The tendency in favor of early rehabilitation in the duration of mechanical ventilation and suggests that early rehabilitation might have the potential to improve short-term survival, and to maintain and increase muscle strength.

However, initiation of rehabilitation in acute phase may make the patients in the ICU

For peer review only - http://bmjopen.bmj.com/site/about/guidelines.xhtml

exhausted with tremendous physical stress, thus increasing ICU mortality [41]. Even though several studies included in the present meta-analysis have described the safety of early physiotherapy in the ICU [29 31-34 42], only two outcomes regarding the safety of early rehabilitation, namely blood lactate level [31] and termination rate of early physiotherapy [33], were evaluated between two groups. Therefore, conclusions should be addressed with caution.

Limitations of the review

The current study has several limitations. First, a small number of patients was included in each outcome. Although Schweickert et al. reported data about delirium, we could not extract cognitive-related outcomes because there were no direct data about delirium-free days [32]. Second, studies included in the current systematic review did not measure the domains of PICS beyond hospital discharge. Third, two of the included studies were pilot/feasibility trials and not powered to find differences in physical or cognitive outcomes [29 33]. Fourth, we could not confirm the exact first day of initiation of rehabilitation in the three studies, although "early rehabilitation" was described in the text and we contacted the authors to acquire additional information [30 42]. **Fifth, we could not find any data regarding direct cognitive assessment in the selected papers. Although cognitive impairment at hospital discharge may not predict long-term cognitive impairment [43], Pandharipande et al. reported that longer durations of delirium on admission was associated with worse global cognition and executive function scores at 3 and 12 months in the surgical ICU. [44] . Therefore, we believe that the assessment of delirium in the ICU and hospital is very important to predict long-term cognitive impairment post-discharge.** Although there are several limitations, the meta-analysis is useful to clarify the effectiveness of early rehabilitation for the prevention of PICS in survivors of critical illness.

For peer review only - http://bmjopen.bmj.com/site/about/guidelines.xhtml

CONCLUSION

Early rehabilitation has **a limited effect on the prevention of PICS, with a significant improvement of short-term physical-related outcomes including MRC score and incidence of ICU-AW, but not cognitive function and mental health-related outcomes or mortality in critically ill patients.** Further large rigorous RCTs are needed to confirm these results.

For peer review only

For peer review only - http://bmjopen.bmj.com/site/about/guidelines.xhtml

Contributors

YK, RF, TH, JH, TT, and SI conceived the idea for this systematic review. YK, RF, TH, JH, TT, and SI developed the methodology for the systematic review and KY and ON supervised the methodological process. The manuscript was drafted by YK and SI. RF, TH, JH, TT, and KY and ON revised the manuscript. All authors critically reviewed and approved the final manuscript.

Funding

There was no funding support for this study.

Competing interests

None declared.

Provenance and peer review

Not commissioned; externally peer reviewed.

Data sharing statement Requests for additional details regarding the study protocol may be made by contacting the corresponding author, Dr Shigeaki Inoue (caf55000@gmail.com).

For peer review only

For peer review only - http://bmjopen.bmj.com/site/about/guidelines.xhtml

FIGURE LEGENDS

Figure 1

Flow chart of the process of study identification and inclusion

Figure 2

Risk of bias summary

Figure 3

The effect of early rehabilitation on short-term outcomes in PICS in ICU patients.1) Physical-related outcomes **A.** Incidence of ICU-AW. **B.** Medical Research Council sum score. 2) Cognitive-related outcomes characterized by delirium-free days. 3) Mental status-related outcome characterized by incidence of HAS/HADS. HAS/HADS, Hospital Anxiety and Depression Scale; CI, confidence interval

Figure 4

The effect of early rehabilitation on long-term outcomes in PICS in ICU patients. The effect of early rehabilitation on health-related quality of life (QOL) scores and in ICU patients. 1). Health-related quality of life (QOL) scores calculated from EQ5D (EuroQol 5 Dimensions). 2) **.** SF-36 PF (Medical Outcomes Study 36-Item Short Form Health Survey Physical Function scale).

For peer review only

REFERENCES

1. Bemis-Dougherty AR, Smith JM. What follows survival of critical illness? Physical therapists' management of patients with post-intensive care syndrome. *Phys Ther* 2013;93(2):179-85. doi: 10.2522/ptj.20110429
2. Iwashyna TJ, Ely EW, Smith DM, et al. Long-term cognitive impairment and functional disability among survivors of severe sepsis. *JAMA : the journal of the American Medical Association* 2010;304(16):1787-94. doi: 10.1001/jama.2010.1553
3. Desai SV, Law TJ, Needham DM. Long-term complications of critical care. *Crit Care Med* 2011;39(2):371-9. doi: 10.1097/CCM.0b013e3181fd66e5
4. Needham DM, Davidson J, Cohen H, et al. Improving long-term outcomes after discharge from intensive care unit: report from a stakeholders' conference. *Crit Care Med* 2012;40(2):502-9. doi: 10.1097/CCM.0b013e318232da75
5. Elliott D, Davidson JE, Harvey MA, et al. Exploring the scope of post-intensive care syndrome therapy and care: engagement of non-critical care providers and survivors in a second stakeholders meeting. *Crit Care Med* 2014;42(12):2518-26. doi: 10.1097/CCM.0000000000000525
6. Gielen S, Adams V, Möbius-Winkler S, et al. Anti-inflammatory effects of exercise training in the skeletal muscle of patients with chronic heart failure. *J Am Coll Cardiol* 2003;42(5):861-68. doi: 10.1016/S0735-1097(03)00848-9
7. Nuhr MJ, Pette D, Berger R, et al. Beneficial effects of chronic low-frequency stimulation of thigh muscles in patients with advanced chronic heart failure. *Eur Heart J* 2004;25(2):136-43. doi: 10.1016/j.ehj.2003.09.027
8. Vivodtzev I, Pépin JL, Vottero G, et al. Improvement in quadriceps strenght and dyspnea in daily tasks after 1 month of electrical stimulation in severely deconditioned and malnourished COPD. *Chest* 2006;129(6):1540-48. doi: 10.1378/chest.129.6.1540
9. Laaksonen DE, Atalay M, Niskanen L, et al. Increased resting and exercise-induced oxidative stress in young IDDM men. *Diabetes care* 1996;19(6):569-74.
10. Mercken EM, Hageman GJ, Schols AMWJ, et al. Rehabilitation decreases exercise-induced oxidative stress in chronic obstructive pulmonary disease. *American Journal of Respiratory and Critical Care Medicine* 2005;172(8):994-1001. doi: 10.1164/rccm.200411-1580OC
11. Nishiyama Y, Ikeda H, Haramaki N, et al. Oxidative stress is related to exercise intolerance in patients with heart failure. *Am Heart J* 1998;135(1):115-20. doi: 10.1016/S0002-8703(98)70351-5

For peer review only

For peer review only - http://bmjopen.bmj.com/site/about/guidelines.xhtml

12. Vincent HK, Morgan JW, Vincent KR. Obesity Exacerbates Oxidative Stress Levels after Acute Exercise. *Med Sci Sports Exerc* 2004;36(5):772-79.

13. Moher D, Booth A, Stewart L. How to reduce unnecessary duplication: use PROSPERO. *BJOG : an international journal of obstetrics and gynaecology* 2014;121(7):784-6. doi: 10.1111/1471-0528.12657

14. Moher D, Shamseer L, Clarke M, et al. Preferred reporting items for systematic review and meta-analysis protocols (PRISMA-P) 2015 statement. *Systematic reviews* 2015;4:1. doi: 10.1186/2046-4053-4-1

15. Shamseer L, Moher D, Clarke M, et al. Preferred reporting items for systematic review and meta-analysis protocols (PRISMA-P) 2015: elaboration and explanation. *BMJ* 2015;349:g7647. doi: 10.1136/bmj.g7647

16. Liberati A, Altman DG, Tetzlaff J, et al. The PRISMA statement for reporting systematic reviews and meta-analyses of studies that evaluate health care interventions: explanation and elaboration. *J Clin Epidemiol* 2009;62(10):e1-34. doi: 10.1016/j.jclinepi.2009.06.006

17. Moher D, Liberati A, Tetzlaff J, et al. Preferred reporting items for systematic reviews and meta-analyses: the PRISMA statement. *PLoS medicine* 2009;6(7):e1000097. doi: 10.1371/journal.pmed.1000097

18. Kondo Y, Fuke R, Hifumi T, et al. Early rehabilitation for the prevention of postintensive care syndrome in critically ill patients: a study protocol for a systematic review and meta-analysis. *BMJ open* 2017;7(3):e013828. doi: 10.1136/bmjopen-2016-013828

19. Paternostro-Sluga T, Grim-Stieger M, Posch M, et al. Reliability and validity of the Medical Research Council (MRC) scale and a modified scale for testing muscle strength in patients with radial palsy. *Journal of rehabilitation medicine* 2008;40(8):665-71. doi: 10.2340/16501977-0235

20. Yang ZZ, Grote DM, Xiu B, et al. TGF-beta upregulates CD70 expression and induces exhaustion of effector memory T cells in B-cell non-Hodgkin's lymphoma. *Leukemia : official journal of the Leukemia Society of America, Leukemia Research Fund, UK* 2014;28(9):1872-84. doi: 10.1038/leu.2014.84

21. Mozzi A, Meregaglia M, Lazzaro C, et al. A comparison of EuroQol 5-Dimension health-related utilities using Italian, UK, and US preference weights in a patient sample. *Clinicoecon Outcomes Res* 2016;8:267-74. doi: 10.2147/CEOR.S98226

22. Obradovic M, Lal A, Liedgens H. Validity and responsiveness of EuroQol-5 dimension (EQ-5D) versus Short Form-6 dimension (SF-6D) questionnaire in chronic pain. *Health Qual Life Outcomes* 2013;11:110. doi: 10.1186/1477-7525-11-110

For peer review only

For peer review only - http://bmjopen.bmj.com/site/about/guidelines.xhtml

23. Yang X, Fan D, Xia Q, et al. The health-related quality of life of ankylosing spondylitis patients assessed by SF-36: a systematic review and meta-analysis. *Qual Life Res* 2016;25(11):2711-23. doi: 10.1007/s11136-016-1345-z

24. JPT H. Chapter 8: Assessing risk of bias in included studies. 2011

25. Duan EH, Oczkowski SJ, Belley-Cote E, et al. beta-Blockers in sepsis: protocol for a systematic review and meta-analysis of randomised control trials. *BMJ open* 2016;6(6):e012466. doi: 10.1136/bmjopen-2016-012466

26. Klotz R, Klaiber U, Grummich K, et al. Percutaneous versus surgical strategy for tracheostomy: protocol for a systematic review and meta-analysis of perioperative and postoperative complications. *Systematic reviews* 2015;4:105. doi: 10.1186/s13643-015-0092-5

27. Davey Smith G, Egger M. Meta-analyses of randomised controlled trials. *Lancet* 1997;350(9085):1182.

28. Sterne JA, Sutton AJ, Ioannidis JP, et al. Recommendations for examining and interpreting funnel plot asymmetry in meta-analyses of randomised controlled trials. *BMJ* 2011;343:d4002. doi: 10.1136/bmj.d4002

29. Brummel NE, Girard TD, Ely EW, et al. Feasibility and safety of early combined cognitive and physical therapy for critically ill medical and surgical patients: the Activity and Cognitive Therapy in ICU (ACT-ICU) trial. *Intensive Care Med* 2014;40(3):370-9. doi: 10.1007/s00134-013-3136-0

30. Jones C, Eddleston J, McCairn A, et al. Improving rehabilitation after critical illness through outpatient physiotherapy classes and essential amino acid supplement: A randomized controlled trial. *Journal of critical care* 2015 doi: 10.1016/j.jcrc.2015.05.002

31. Kayambu G, Boots R, Paratz J. Early physical rehabilitation in intensive care patients with sepsis syndromes: a pilot randomised controlled trial. *Intensive Care Med* 2015;41(5):865-74. doi: 10.1007/s00134-015-3763-8

32. Schweickert WD, Pohlman MC, Pohlman AS, et al. Early physical and occupational therapy in mechanically ventilated, critically ill patients: a randomised controlled trial. *Lancet* 2009;373(9678):1874-82. doi: 10.1016/S0140-6736(09)60658-9

33. Hodgson CL, Bailey M, Bellomo R, et al. A Binational Multicenter Pilot Feasibility Randomized Controlled Trial of Early Goal-Directed Mobilization in the ICU. *Crit Care Med* 2016;44(6):1145-52. doi: 10.1097/CCM.0000000000001643

34. Morris PE, Berry MJ, Files DC, et al. Standardized Rehabilitation and Hospital Length of Stay Among Patients With Acute Respiratory Failure: A Randomized Clinical Trial. *JAMA : the*

For peer review only

For peer review only - http://bmjopen.bmj.com/site/about/guidelines.xhtml

journal of the American Medical Association 2016;315(24):2694-702. doi: 10.1001/jama.2016.7201

35. Stiller K. Physiotherapy in intensive care: an updated systematic review. *Chest* 2013;144(3):825-47. doi: 10.1378/chest.12-2930

36. Kayambu G, Boots R, Paratz J. Physical therapy for the critically ill in the ICU: a systematic review and meta-analysis. *Crit Care Med* 2013;41(6):1543-54. doi: 10.1097/CCM.0b013e31827ca637

37. Tipping CJ, Harrold M, Holland A, et al. The effects of active mobilisation and rehabilitation in ICU on mortality and function: a systematic review. *Intensive Care Med* 2016 doi: 10.1007/s00134-016-4612-0

38. Castro-Avila AC, Seron P, Fan E, et al. Effect of Early Rehabilitation during Intensive Care Unit Stay on Functional Status: Systematic Review and Meta-Analysis. *PloS one* 2015;10(7):e0130722. doi: 10.1371/journal.pone.0130722

39. Denehy L, Skinner EH, Edbrooke L, et al. Exercise rehabilitation for patients with critical illness: a randomized controlled trial with 12 months of follow-up. *Crit Care* 2013;17(4):R156. doi: 10.1186/cc12835 [published Online First: 2013/07/26]

40. Moss M, Nordon-Craft A, Malone D, et al. A Randomized Trial of an Intensive Physical Therapy Program for Patients with Acute Respiratory Failure. *Am J Respir Crit Care Med* 2016;193(10):1101-10. doi: 10.1164/rccm.201505-1039OC

41. Greening NJ, Williams JE, Hussain SF, et al. An early rehabilitation intervention to enhance recovery during hospital admission for an exacerbation of chronic respiratory disease: randomised controlled trial. *BMJ* 2014;349:g4315. doi: 10.1136/bmj.g4315

42. Cameron JI, Chu LM, Matte A, et al. One-Year Outcomes in Caregivers of Critically Ill Patients. *The New England journal of medicine* 2016;374(19):1831-41. doi: 10.1056/NEJMoa1511160

43. Woon FL, Dunn CB, Hopkins RO. Predicting cognitive sequelae in survivors of critical illness with cognitive screening tests. *Am J Respir Crit Care Med* 2012;186(4):333-40. doi: 10.1164/rccm.201112-2261OC

44. Pandharipande PP, Girard TD, Jackson JC, et al. Long-term cognitive impairment after critical illness. *The New England journal of medicine* 2013;369(14):1306-16. doi: 10.1056/NEJMoa1301372

For peer review only

For peer review only - http://bmjopen.bmj.com/site/about/guidelines.xhtml

Table 1. Characteristics of included studies

Source	Population	No. of patients			Age (years)		APACHE II score		Intervention		Timing of first rehabilitation/ standard care	Follow-up	Outcome		Ref
		Total	Early rehabilitation	Control	Early rehabilitation	Control	Early rehabilitation	Control	Early rehabilitation	Control			Short-term (during the hospitalization)	Long-term (post-discharge)	
Brummel 2014	Respiratory failure and/or septic, cardiogenic, or haemorrhagic shock	44	22	22	62 [48–67]	60 [51–69]	21.5 [20.0–28.8]	27.0 [17.5–31.0]	Passive range of motion exercises to independent ambulation, guided by the patient's RASS (daily). For RASS -1 to +1: active exercises, sitting at the edge of bed, standing/transferring, ADL training, and walking	Physical therapy after it was ordered by the treating clinicians (1–2 per week).	Intervention: 1 (1–1), control: 3 (2–6)	3 months	ventilator-free days, delirium-free days,	EQ5D,	29
Hodgson 2016	Critically ill adults mechanically ventilated for more than 24 hours	50	29	21	64 ± 12	53 ± 15	19.8 ± 9.8	15.9 ± 6.9	Functional rehabilitation treatment conducted at the highest level of activity possible for that patient assessed by the ICU mobility scale	Not protocolised and all usual unit practice was continued, with no restrictions on physical therapy or sedation practice	Intervention: 3 (2–4), control: 3 (2–4)	6 months	MRC, ICU-AW, mechanical ventilation days,	EQ5D,	33
Jones 2015	Patients should be aged 45 years or older, had a combined ICU and pre-ICU stay of 5 days or more	42	22	20	64 ± 13	60 ± 12	17 ± 10	14 ± 4	In addition to the self-help program, a 6-week program of supervised physiotherapy sessions	Patient-controlled self-help rehabilitation program	N/A	3 months	ventilator-free days, HAS/HADS		30
Kayambu 2015	Participants older than18 years who remained mechanically ventilated for more than 48 hours and diagnosed with sepsis	50	26	24	62.5 [30–83]	65.5 [37–85]	28 ± 7.6	27 ± 6.8	Electrical muscle stimulation, passive range of motion, active range of motion, sitting out of bed, transfers, ambulation for 30 minutes, one to two times daily	Standard ICU care, which included physical therapy	Within 48 hours of sepsis	6 months	MRC, ventilator-free days, HAS/HADS,	SF36-PF,	31
Morris	Acute respiratory	300	150	150	55 ± 17	58 ±	N/A	N/A	Passive range of	Physical therapy	Intervention:	6	ventilat	SF36-	34

For peer review only

2016	failure patients requiring mechanical ventilation					14			motion, physical therapy, and progressive resistance exercises for 7 days per week	weekday when ordered by the clinical team	1 (0–2), control: 7 (4–10)	month s	or-free days, deliriu m-free days,	PF	
Schwei ckert 2009	Patients who had been on mechanical ventilation for less than 72 hours	104	49	55	57.7 [36.3–69.1]	54.4 [46.5–66.4]	20 [15.8–24]	19 [13.3–23]	Early exercise and physical and occupational therapy on the day of enrolment	Therapy as ordered by the primary care team	Intervention: 1.5 (1.0–2.1), control: 7.4 (6.0–10.9)	N/A	MRC, ICU-AW, ventilat or-free days, deliriu m days,		32

For peer review only

For peer review only - http://bmjopen.bmj.com/site/about/guidelines.xhtml

Table 2

Quality assessment and summary of findings for the main comparison

Quality assessment							№ of patients		Effect		Quality	Importance
№ of studies	Study design	Risk of bias	Inconsistency	Indirectness	Imprecision	Other considerations	Early rehabilitation	Control	Relative (95% CI)	Absolute (95% CI)		
Incidence of ICU-AW												
2	randomised trials	serious [a]	not serious	not serious	serious [b]	none	22/78 (28.2%)	37/76 (48.7%)	**OR 0.42** (0.22 to 0.82)	**202 fewer per 1,000** (from 49 fewer to 314 fewer)	⨁⨁◯◯ LOW	CRITICAL
MRC												
3	randomised trials	serious [a]	not serious	not serious	serious [b]	none	97	99	-	SMD **0.38 higher** (0.1 higher to 0.66 higher)	⨁⨁◯◯ LOW	CRITICAL
Delirium-free days												
3	randomised trials	serious [a]	not serious	not serious	serious [b]	none	164	162	-	SMD **0.02 lower** (0.23 lower to 0.2 higher)	⨁⨁◯◯ LOW	CRITICAL
Incidence of HADS/HAS												
2	randomised trials	serious [a]	serious	not serious	serious [b]	none	10/48 (20.8%)	11/44 (25.0%)	**OR 0.79** (0.29 to 2.12)	**42 fewer per 1,000** (from 162 fewer to 164 more)	⨁◯◯◯ VERY LOW	CRITICAL
Health related QOL score (EQ5D)												
2	randomised trials	serious [a]	serious [c]	not serious	serious [b]	none	35	28	-	SMD **0.11 SD higher** (0.86 lower to 1.09 higher)	⨁◯◯◯ VERY LOW	CRITICAL
SF36-PF												
2	randomised trials	serious [a]	not serious	not serious	serious [b]	none	93	98	-	SMD **2.41 higher** (0.75 lower to 5.58 higher)	⨁⨁◯◯ LOW	CRITICAL

CI: Confidence interval; **SMD:** Standardized mean difference; **OR:** Odds ratio

a. Most of the studies are classified as unclear or high risk of bias (performance bias and attrition bias)

b. Confidence intervals for absolute effects are wide and include no effect and important harm and/or small sample size (The number of events is <300)

c. Substantial heterogeneity present

GRADE Working Group grades of evidence High quality: further research is very unlikely to change our confidence in the estimate of effect. Moderate quality: further research is likely to have an important impact on our confidence in the estimate of effect and may change the estimate. Low quality: further research is very likely to have an important impact on our confidence in the estimate of effect and is likely to change the estimate. Very low quality: we are very uncertain about the estimate.

For peer review only

Figure 1

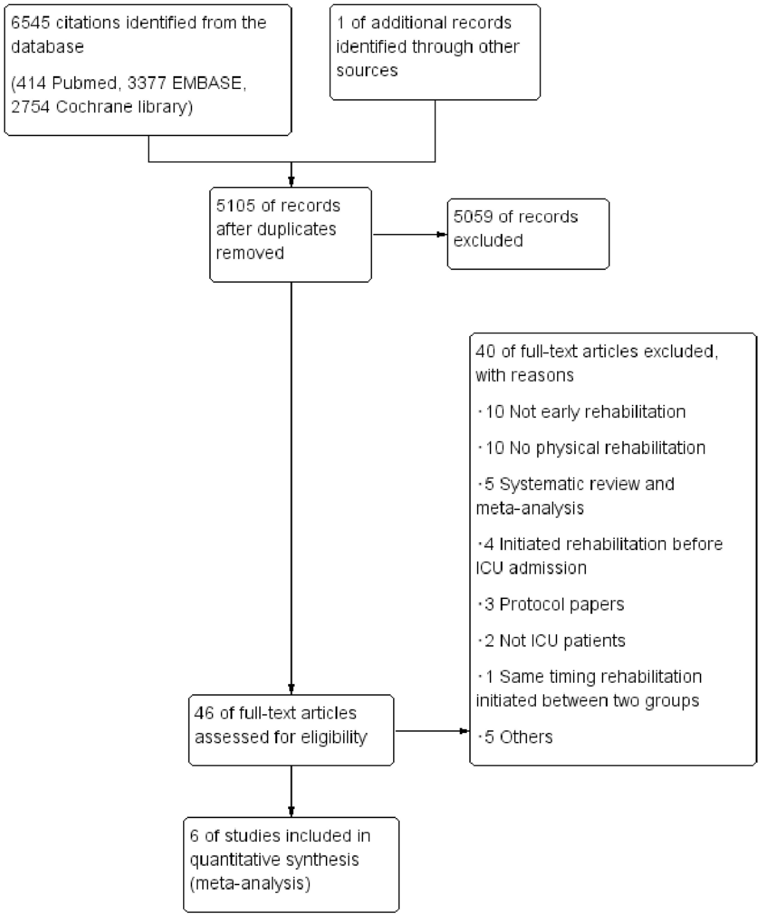

Figure 1

254x338mm (300 x 300 DPI)

Figure 2

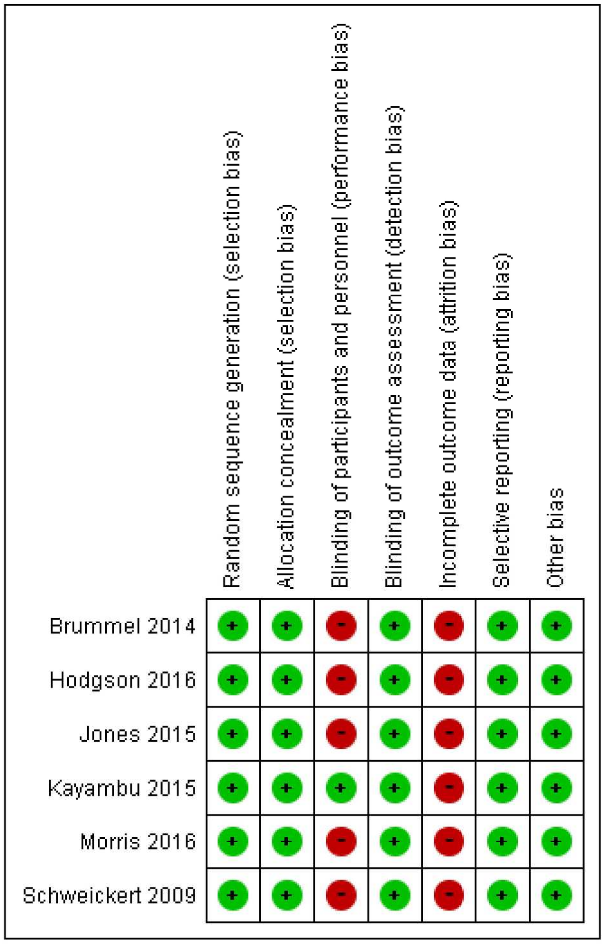

Figure 2

254x338mm (300 x 300 DPI)

Figure 3

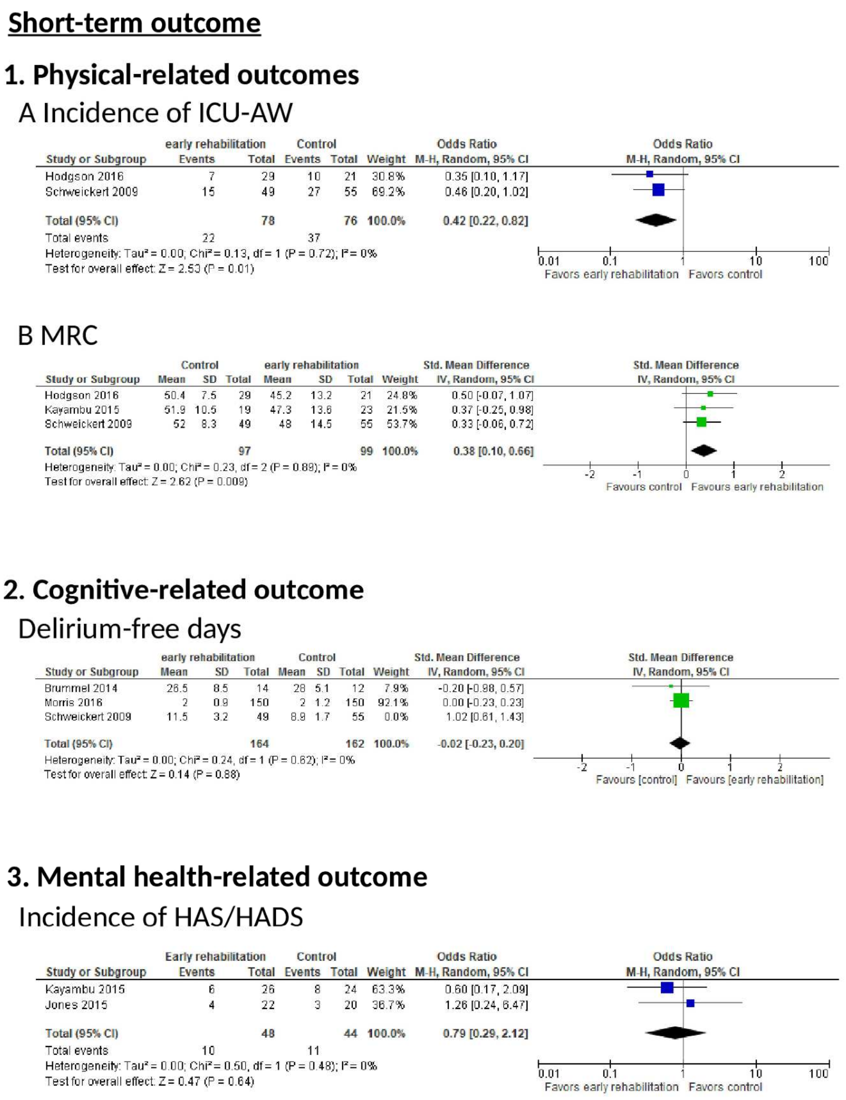

Figure 3

254x338mm (300 x 300 DPI)

Figure 4

Long-term outcome

1 Health-related QOL scores

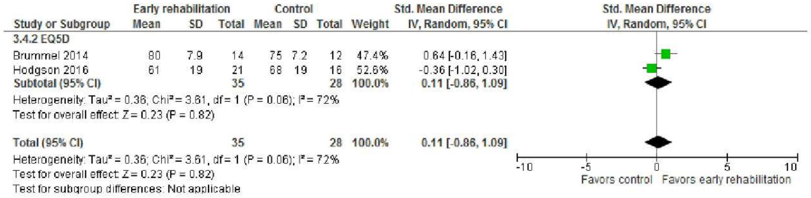

2 SF-36PF

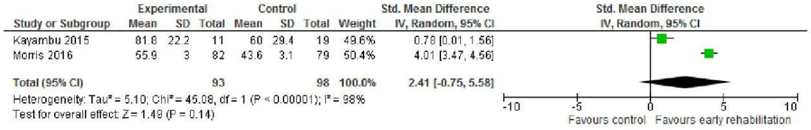

Figure 4

254x338mm (300 x 300 DPI)

PRISMA 2009 Checklist

Section/topic	#	Checklist item	Reported on page #
**TITLE**			
Title	1	Identify the report as a systematic review, meta-analysis, or both.	1
**ABSTRACT**			
Structured summary	2	Provide a structured summary including, as applicable: background; objectives; data sources; study eligibility criteria, participants, and interventions; study appraisal and synthesis methods; results; limitations; conclusions and implications of key findings; systematic review registration number.	3
**INTRODUCTION**			
Rationale	3	Describe the rationale for the review in the context of what is already known.	5
Objectives	4	Provide an explicit statement of questions being addressed with reference to participants, interventions, comparisons, outcomes, and study design (PICOS).	5
**METHODS**			
Protocol and registration	5	Indicate if a review protocol exists, if and where it can be accessed (e.g., Web address), and, if available, provide registration information including registration number.	6
Eligibility criteria	6	Specify study characteristics (e.g., PICOS, length of follow-up) and report characteristics (e.g., years considered, language, publication status) used as criteria for eligibility, giving rationale.	6, 7
Information sources	7	Describe all information sources (e.g., databases with dates of coverage, contact with study authors to identify additional studies) in the search and date last searched.	6, 8
Search	8	Present full electronic search strategy for at least one database, including any limits used, such that it could be repeated.	Suppl 1
Study selection	9	State the process for selecting studies (i.e., screening, eligibility, included in systematic review, and, if applicable, included in the meta-analysis).	7, 8, 9
Data collection process	10	Describe method of data extraction from reports (e.g., piloted forms, independently, in duplicate) and any processes for obtaining and confirming data from investigators.	8
Data items	11	List and define all variables for which data were sought (e.g., PICOS, funding sources) and any assumptions and simplifications made.	-
Risk of bias in individual studies	12	Describe methods used for assessing risk of bias of individual studies (including specification of whether this was done at the study or outcome level), and how this information is to be used in any data synthesis.	7, 8
Summary measures	13	State the principal summary measures (e.g., risk ratio, difference in means).	8, 9
Synthesis of results	14	Describe the methods of handling data and combining results of studies, if done, including measures of consistency (e.g., I^2) for each meta-analysis.	8, 9

For peer review only - http://bmjopen.bmj.com/site/about/guidelines.xhtml

PRISMA 2009 Checklist

Section/topic	#	Checklist item	Reported on page #
Risk of bias across studies	15	Specify any assessment of risk of bias that may affect the cumulative evidence (e.g., publication bias, selective reporting within studies).	9
Additional analyses	16	Describe methods of additional analyses (e.g., sensitivity or subgroup analyses, meta-regression), if done, indicating which were pre-specified.	-
**RESULTS**			
Study selection	17	Give numbers of studies screened, assessed for eligibility, and included in the review, with reasons for exclusions at each stage, ideally with a flow diagram.	10, Figure 1
Study characteristics	18	For each study, present characteristics for which data were extracted (e.g., study size, PICOS, follow-up period) and provide the citations.	Table 1
Risk of bias within studies	19	Present data on risk of bias of each study and, if available, any outcome level assessment (see item 12).	Figure 2
Results of individual studies	20	For all outcomes considered (benefits or harms), present, for each study: (a) simple summary data for each intervention group (b) effect estimates and confidence intervals, ideally with a forest plot.	Figure 3, 4, 5
Synthesis of results	21	Present results of each meta-analysis done, including confidence intervals and measures of consistency.	11, 12, 13, Figure 3
Risk of bias across studies	22	Present results of any assessment of risk of bias across studies (see Item 15).	11, 12, 13, Figure 3
Additional analysis	23	Give results of additional analyses, if done (e.g., sensitivity or subgroup analyses, meta-regression [see Item 16]).	-
**DISCUSSION**			
Summary of evidence	24	Summarize the main findings including the strength of evidence for each main outcome; consider their relevance to key groups (e.g., healthcare providers, users, and policy makers).	14
Limitations	25	Discuss limitations at study and outcome level (e.g., risk of bias), and at review-level (e.g., incomplete retrieval of identified research, reporting bias).	16
Conclusions	26	Provide a general interpretation of the results in the context of other evidence, and implications for future research.	17
**FUNDING**			
Funding	27	Describe sources of funding for the systematic review and other support (e.g., supply of data); role of funders for the systematic review.	18

For peer review only - http://bmjopen.bmj.com/site/about/guidelines.xhtml

PRISMA 2009 Checklist

From: Moher D, Liberati A, Tetzlaff J, Altman DG, The PRISMA Group (2009). Preferred Reporting Items for Systematic Reviews and Meta-Analyses: The PRISMA Statement. PLoS Med 6(6): e1000097. doi:10.1371/journal.pmed1000097

For more information, visit: **www.prisma-statement.org**.

For peer review only

BMJ Open

Early rehabilitation to prevent post-intensive care syndrome in critically ill patients: a systematic review and meta-analysis

Journal:	*BMJ Open*
Manuscript ID	bmjopen-2017-019998.R1
Article Type:	Research
Date Submitted by the Author:	06-Dec-2017
Complete List of Authors:	Fuke, Ryota; Tohoku Ika Yakka Daigaku Wakabayashi Byoin<br>Hifumi, Toru; Kagawa University Hospital, Emergency Medical Center<br>Kondo, Yutaka; Ryukyu Daigaku,<br>Hatakeyama, Junji; Yokohama Shiritsu Minato Sekijuji Byoin<br>Takei, Tetsuhiro; Yokohama Shiritsu Minato Sekijuji Byoin, Intensive Care Unit<br>Yamakawa, Kazuma; Osaka General Medical Center, Department of Emergency and Critical Care<br>Inoue, Shigeaki; Tokai University Hachioji city Hospital, Department of Emergency and Critical Care Medicine<br>Nishida, Osamu; Fujita Hoken Eisei Daigaku Igakubu Daigakuin Igaku Kenkyuka
**Primary Subject Heading**:	Emergency medicine
Secondary Subject Heading:	Rehabilitation medicine
Keywords:	early rehabilitation, intensive care unit, post-intensive care syndrome, sepsis

SCHOLARONE™
Manuscripts

Early rehabilitation to prevent post-intensive care syndrome in critically ill patients: a systematic review and meta-analysis

Ryota Fuke, MD[1*], Toru Hifumi, MD[2*], Yutaka Kondo, MD, PhD[3], Junji Hatakeyama, MD[4], Tetsuhiro Takei, MD, PhD[4], Kazuma Yamakawa, MD, PhD[5], Shigeaki Inoue, MD, PhD, MSCI[6], Osamu Nishida, MD, PhD[7]

[1] Division of Infectious Diseases and Infection Control, Tohoku Medical and Pharmaceutical University, Sendai City, Miyagi, JAPAN

[2] Emergency Medical Center, Kagawa University Hospital, Kita-gun-Takamatsu, JAPAN

[3] Department of Emergency and Critical Care Medicine, Graduate School of Medicine, University of the Ryukyus, Nakagami-gun, Okinawa, JAPAN

[4] Department of Intensive Care Medicine, Yokohama City Minato Red Cross Hospital, Yokohama City, Kanagawa, JAPAN

[5] Division of Trauma and Surgical Critical Care, Osaka General Medical Center, Osaka City, Osaka, JAPAN

[6] Department of Emergency and Critical Care Medicine, Tokai University Hachioji Hospital, Hachioji, Tokyo, JAPAN

[7] Department of Anaesthesiology and Critical Care Medicine, Fujita Health University School of Medicine, Toyoake City, Aichi, JAPAN

* Ryota Fuke and Toru Hifumi contributed equally to this manuscript.

Address correspondence and reprint requests to: Shigeaki Inoue, Department of Emergency and Critical Care Medicine, Tokai University Hachioji Hospital, Ishikawa-cho 1838, Hachioji City,

For peer review only

Tokyo 192-0032, Japan. Tel.: +81-42-639-1111; E-mail caf55000@gmail.com

Running title: Early rehabilitation to prevent post-intensive care syndrome

Keywords: early rehabilitation, intensive care unit, post-intensive care syndrome, sepsis

Conflict of interest: The authors declare that they have no conflicts of interest.

Word count: 3032 words

Number of tables: 2

Number of figures: 4

Number of supplementary files for online-only publication: 2

Number of references: 42

For peer review only

ABSTRACT

Introduction: We examined the effectiveness of early rehabilitation for the prevention of post-intensive care syndrome (PICS), characterised by an impaired physical, cognitive, or mental health status, among survivors of critical illness. **Methods**: We performed a systematic literature search of several databases (Medline, Embase, and Cochrane Central Register of Controlled Trials) and a manual search to identify randomised controlled trials (RCTs) comparing the effectiveness of early rehabilitation versus no early rehabilitation or standard care for the prevention of PICS. The primary outcomes were short-term physical-, cognitive-, and mental health-related outcomes assessed during hospitalisation. The secondary outcomes were the standardised, long-term health-related quality of life scores (EuroQol 5 Dimension [EQ5D] and the Medical Outcomes Study 36-Item Short Form Health Survey Physical Function scale [SF-36 PF]). We used the Grading of Recommendations Assessment, Development and Evaluation (GRADE) approach to rate the quality of evidence (QoE). **Results**: Six RCTs selected from 5,105 screened abstracts were included. Early rehabilitation significantly improved short-term physical-related outcomes, as indicated by an increased Medical Research Council scale score (MRC score) [standardised mean difference (SMD): 0.38, 95% confidence interval (CI): 0.10–0.66, p = 0.009] (QoE: low) and a decreased incidence of ICU-acquired weakness (ICU-AW) [odds ratio (OR) 0.42, 95% CI 0.22 to 0.82, p = 0.01] (QoE: low), compared to standard care or no early rehabilitation. However, the two groups did not differ in terms of cognitive-related delirium-free days (SMD: -0.02, 95% CI: -0.23–0.20) (QoE: low) and the mental health-related Hospital Anxiety and Depression Scale score (odds ratio: 0.79, 95% CI: 0.29–2.12) (QoE: low). Early rehabilitation did not improve the long-term outcomes of PICS as characterised by EQ5D and SF-36PF. **Conclusions**: Early rehabilitation improved only short-term physical-related outcomes in critically ill patients. Additional large RCTs are needed.

For peer review only

For peer review only - http://bmjopen.bmj.com/site/about/guidelines.xhtml

Strengths and limitations of this meta-analysis

- ✓ This meta-analysis revealed a limited effect of early rehabilitation on the prevention of PICS; although early rehabilitation significantly improved short-term physical-related outcomes in critically ill patients, it did not affect cognitive and mental status-related outcomes.
- ✓ This meta-analysis was mainly limited by (1) the small number of patients in the included RCTs; (2) a lack of detailed analysis of the adverse effects and PICS domains after hospital discharge; and (3) no confirmation of the exact first day of rehabilitation initiation in three of the eight included RCTs.
- ✓ This meta-analysis clarifies the effectiveness and limitations of early rehabilitation for the prevention of PICS in survivors of critical illness.

For peer review only

For peer review only - http://bmjopen.bmj.com/site/about/guidelines.xhtml

INTRODUCTION

Dramatic developments and improvements in the technique, instruments, and education systems used in intensive care units (ICUs) have reduced the mortality of critically ill patients over the past four decades [1]. However, this evolution of life-saving interventions has led to increasing numbers of surviving critically ill patients with an impaired ability to return to physical and mental aspects of normal life [2]. These persistent physical, cognitive and mental impairments experienced by ICU survivors present serious obstacles to discharge from the hospital to a home setting and, once home, to a return to normal daily life [2 3].

Post-intensive care syndrome (PICS) was established as a new syndrome encompassing new or worsening impairments in physical, cognitive, or mental health status that arise after critical illness and persist beyond acute care hospitalisation, with the aim initiating improvements for ICU survivors and their families across the continuum of care [4]. Since the establishment of PICS in 2010, observational studies have evaluated the independent factors associated with this syndrome [5]; however, few intervention studies have targeted the prevention of PICS.

Physiotherapy with early rehabilitation is considered an integral component of the multidisciplinary management of patients in ICUs. In other populations, exercise has been shown to improve strength and function, decrease inflammation [6-8], and affect oxidative stress [9-12], leading to suggestions that early physiotherapy may prevent or reverse some physical impairments in ICU patients. However, no systematic review has investigated the effectiveness of early rehabilitation for the prevention of PICS in ICU patients. The present systematic review aimed to assess the effectiveness of early rehabilitative interventions for the prevention of PICS in ICU patients.

For peer review only - http://bmjopen.bmj.com/site/about/guidelines.xhtml

METHODS

Protocols and registration

This review protocol was registered in PROSPERO, an International Prospective Register of Systematic Reviews at the National Institute for Health Research and Centre for Reviews and Dissemination (CRD) at the University of York (http://www.crd.york.ac.uk/PROSPERO/) (Registration No. CRD42016039759) [13]. This protocol adhered to the Preferred Reporting Items for Systematic Reviews and Meta-Analyses Protocols (PRISMA) statements [14 15], and the systematic review was reported according to PRISMA guidelines [14 16 17]. The protocol for this systematic review was previously published [18].

Search strategy

We searched the Medline (via PubMed from 1996 to 7 June 2016), Embase (until 7 June 2016), and Cochrane Central Register of Controlled Trials (CENTRAL) databases (until 7 June 2016) for full-text clinical trials conducted in humans to retrieve relevant articles for the literature review. We also performed a manual search to retrieve all potentially relevant articles from 8 June to 31 August, 2016. The key search terms used to identify potential studies are listed in Supplementary file 1.

Study selection and inclusion criteria

Three authors (YK, RF, SI) performed the first-line comprehensive literature search and removal of duplicates. Subsequently, two randomly selected authors independently screened the study titles and abstracts for potential relevance. The kappa value for selection bias was 0.396. When disagreements arose between reviewers, the full text of the paper was retrieved, and the disagreements were reconsidered and discussed until a consensus was reached. The full texts of articles included in the final selection were reviewed by two randomly chosen authors. We included trials with the

For peer review only - http://bmjopen.bmj.com/site/about/guidelines.xhtml

following characteristics:

Study types: Randomised controlled trials (RCTs) were included; non-randomised and observational studies were excluded.

Population: Adult patients (aged >18 years) admitted to the ICU were included. We excluded patients with traumatic brain injury and stroke who did not fulfil the PICS criteria, specifically those with acquired physical and psychiatric/cognitive dysfunction after ICU admission.

Intervention: The intervention was early rehabilitation, defined as (1) starting at an earlier time point than usual care or (2) administration within 7 days of ICU admission. 'Rehabilitation' included all physiotherapy, occupational therapy, and palliative care-related support. We excluded RCTs in which rehabilitation was initiated before ICU admission and those comparing early rehabilitation with another intervention.

Control: The control group received standard care or no early rehabilitation.

Outcome: The primary outcomes (i.e., short-term outcomes assessed during hospitalisation) were as follows: (1) physical-related outcomes [incidence of ICU-acquired weakness (AW), Medical Research Council (MRC) [19] scale score], (2) cognitive-related outcomes (delirium-free days); and (3) mental status-related outcomes [Hospital Anxiety and Depression Scale (HADS)] [20]. The secondary outcomes (long-term outcomes assessed post-discharge) included the standardised Health-Related Quality of Life with EuroQol 5 Dimensions (EQ5D) test [21 22] and the Medical Outcomes Study 36-Item Short Form Health Survey Physical Function scale (SF-36 PF) [23] as measures of long-term physical function.

Assessment of the risk of bias

To assess the quality of the included studies, we adopted the Cochrane risk of bias tool [24]. Two randomly selected authors performed the risk of bias assessment; disagreements were resolved by

For peer review only - http://bmjopen.bmj.com/site/about/guidelines.xhtml

discussion, with the inclusion of a third reviewer if necessary. We considered the risk of bias for each element to be 'high' when bias was present and likely to affect outcomes, and 'low' when bias was not present or present but unlikely to affect outcomes [25].

Rating the quality of evidence (QoE) using the GRADE approach

Two authors (RF and TH) independently used the Grading of Recommendations Assessment, Development, and Evaluation (GRADE) tool to rate the quality of evidence (QoE). We used the GRADE approach to rate the QoE of early rehabilitation for outcomes. Although the QoE represents a continuum, we assessed the quality of the body of evidence for each outcome categorised as high, moderate, low, or very low using the GRADE pro Guideline Development Tool.

Data extraction and management

The author(s), title, journal name, year of publication, website URL, and abstract of each included article were identified. Conference abstracts were excluded. Data from each study were extracted independently by two review authors. When faced with insufficient or incomplete data, we contacted the authors of the studies directly. Information related to the study population, sample size, interventions, comparators, potential biases in the conduct of the trial, outcomes (including adverse events), follow-up, and statistical analysis methods were extracted from the original reports into specially designed paper forms and then entered into a spreadsheet.

Summarisation of data and treatment effects

As data from several trials were available, we performed a meta-analysis according to the Cochrane Handbook for Systematic Reviews of Interventions and PRISMA guidelines using Review Manager software (RevMan 5.3, Copenhagen, Denmark: The Nordic Cochrane Centre, the Cochrane

For peer review only - http://bmjopen.bmj.com/site/about/guidelines.xhtml

Collaboration 2014). We summarised the results of this meta-analysis using the generic inverse variance method to facilitate the pooling of estimates of treatment effects. We used odds ratios (ORs) with 95% confidence intervals (CIs) for dichotomous outcomes, and mean differences or standardised mean differences (SMDs) with 95% CIs for continuous outcomes when appropriate. If a quantitative synthesis was not appropriate for a particular outcome, we devised a qualitative summary of that outcome.

Assessment of heterogeneity

For each outcome, we assessed heterogeneity between trials using I^2 statistics to quantify inconsistencies. We considered that significant heterogeneity was present when the reason for heterogeneity could not be explained and the I^2 was ≥50%. The presence of strong clinical heterogeneity was considered when deciding to perform a quantitative data synthesis or a sensitivity analysis with a special focus [26].

Data synthesis

Estimates were pooled using a random effects model. We performed our analysis based on all published or available data.

For peer review only

RESULTS

Literature search

The PRISMA flow chart of study selection is shown in Figure 1. The combined search strategy identified 5105 citations, of which 46 were judged to be potentially eligible based on the abstract. After excluding 40 of these citations following a full-text review (Supplementary file 2), six RCTs that had enrolled 709 patients were deemed eligible and were included in the review [27-32].

Characteristics of included studies

The study characteristics are summarised in Table 1. Early rehabilitation was initiated within 3 days (median or mean) of ICU admission in five studies [27 29-32]. The first day of rehabilitation was not confirmed in three studies, although early rehabilitation was described in the text, and we contacted the authors to acquire additional information [28]. The rehabilitation strategies administered to each intervention group varied among protocols.

For peer review only - http://bmjopen.bmj.com/site/about/guidelines.xhtml

Table 1. Characteristics of included studies

Source	Population	No. of patients			Age (years)		APACHE II score		Intervention		Timing of first rehabilitation/ standard care	Follo w-up	Outcome		R e f
		To tal	Early rehabilitati on	Cont rol	Early rehabilit ation	Cont rol	Early rehabilitati on	Cont rol	Early rehabilitation	Control			Short-t erm (durin g the hospita lizatio n)	Long -term (post -disc harg e)	
Brum mel 2014	Respiratory failure and/or septic, cardiogenic, or haemorrhagic shock	44	22	22	62 [48– 67]	60 [51– 69]	21.5 [20.0– 28.8]	27.0 [17.5 – 31.0]	Passive range of motion exercises to independent ambulation, guided by the patient's RASS (daily). For RASS -1 to +1: active exercises, sitting at the edge of bed, standing/transferring, ADL training, and walking	Physical therapy after it was ordered by the treating clinicians (1–2 per week).	Intervention: 1 (1–1), control: 3 (2– 6)	3 mont hs	ventila tor-fre e days, deliriu m-free days,	EQ5 D,	2 9
Hodgs on 2016	Critically ill adults mechanically ventilated for more than 24 hours	50	29	21	64 ± 12	53 ± 15	19.8 ± 9.8	15.9 ± 6.9	Functional rehabilitation treatment conducted at the highest level of activity possible for that patient assessed by	Not protocolised and all usual unit practice was continued, with no	Intervention: 3 (2–4), control: 3 (2– 4)	6 mont hs	MRC, ICU-A W, mecha nical ventila	EQ5 D,	3 3

For peer review only - http://bmjopen.bmj.com/site/about/guidelines.xhtml

									the ICU mobility scale	restrictions on physical therapy or sedation practice			tion days,		
Jones 2015	Patients should be aged 45 years or older, had a combined ICU and pre-ICU stay of 5 days or more	42	22	20	64 ± 13	60 ± 12	17 ± 10	14 ± 4	In addition to the self-help program, a 6-week program of supervised physiotherapy sessions	Patient-controll ed self-help rehabilitation program	N/A	3 mont hs	ventila tor-fre e days, HAS/ HADS		3 0
Kaya mbu 2015	Participants older than18 years who remained mechanically ventilated for more than 48 hours and diagnosed with sepsis–	50	26	24	62.5 [30–83]	65.5 [37– 85]	28 ± 7.6	27 ± 6.8	Electrical muscle stimulation, passive range of motion, active range of motion, sitting out of bed, transfers, ambulation for 30 minutes, one to two times daily	Standard ICU care, which included physical therapy	Within 48 hours of sepsis	6 mont hs	MRC, ventila tor-fre e days, HAS/ HADS ,	SF36 -PF,	3 1
Morris 2016	Acute respiratory failure patients requiring mechanical ventilation	30 0	150	150	55 ± 17	58 ± 14	N/A	N/A	Passive range of motion, physical therapy, and progressive resistance exercises for 7 days per week	Physical therapy weekday when ordered by the clinical team	Intervention: 1 (0–2), control: 7 (4– 10)	6 mont hs	ventila tor-fre e days, deliriu m-free days,	SF36 -PF	3 4
Schwe ickert 2009	Patients who had been on mechanical ventilation for less than 72 hours	10 4	49	55	57.7 [36.3– 69.1]	54.4 [46.5 – 66.4]	20 [15.8– 24]	19 [13.3 –23]	Early exercise and physical and occupational therapy on the day of enrolment	Therapy as ordered by the primary care team	Intervention: 1.5 (1.0–2.1), control: 7.4 (6.0–10.9)	N/A	MRC, ICU-A W, ventila tor-fre e days, deliriu m days,		3 2

For peer review only - http://bmjopen.bmj.com/site/about/guidelines.xhtml

For peer review only

For peer review only - http://bmjopen.bmj.com/site/about/guidelines.xhtml

Risk of bias in the included trials

The evaluation of the risk of bias in each included RCT is shown in the risk of bias summary for PICS-related outcomes without in Figure 2. Regarding blinding, eight trials involved open-label RCTs. The blinding of outcome assessments was not described for 42 RCTs. In all RCTs, attrition bias was observed because of a per-protocol analysis, the inclusion of many patients who dropped out of the study, and death. The performance bias of mortality as an outcome measure was low, however, because blinding did not affect mortality.

Outcomes

The quality assessment and a summary of each outcome measure are shown in Table 2.

For peer review only - http://bmjopen.bmj.com/site/about/guidelines.xhtml

Table 2

Quality assessment and summary of findings for the main comparison

Quality assessment							№ of patients		Effect		Quality	Importance
№ of studies	Study design	Risk of bias	Inconsistency	Indirectness	Imprecision	Other considerations	Early rehabiritation	Control	Relative (95% CI)	Absolute (95% CI)		
Incidence of ICU-AW												
2	randomised trials	serious [a]	not serious	not serious	serious [b]	none	22/78 (28.2%)	37/76 (48.7%)	**OR 0.42** (0.22 to 0.82)	**202 fewer per 1,000** (from 49 fewer to 314 fewer)	⊕⊕◯◯ LOW	CRITICAL
MRC												
3	randomised trials	serious [a]	not serious	not serious	serious [b]	none	97	99	-	SMD **0.38 higher** (0.1 higher to 0.66 higher)	⊕⊕◯◯ LOW	CRITICAL
Delirium-free days												

For peer review only

Quality assessment							№ of patients		Effect		Quality	Importance
№ of studies	Study design	Risk of bias	Inconsistency	Indirectness	Imprecision	Other considerations	Early rehabiritation	Control	Relative (95% CI)	Absolute (95% CI)		
3	randomised trials	serious [a]	not serious	not serious	serious [b]	none	164	162	-	SMD **0.02 lower** (0.23 lower to 0.2 higher)	⊕⊕◯◯ LOW	CRITICAL
Incidence of HADS/HAS												
2	randomised trials	serious [a]	serious	not serious	serious [b]	none	10/48 (20.8%)	11/44 (25.0%)	**OR 0.79** (0.29 to 2.12)	**42 fewer per 1,000** (from 162 fewer to 164 more)	⊕◯◯◯ VERY LOW	CRITICAL
Health related QOL score (EQ5D)												
2	randomised trials	serious [a]	serious [c]	not serious	serious [b]	none	35	28	-	SMD **0.11 SD higher** (0.86 lower to 1.09 higher)	⊕◯◯◯ VERY LOW	CRITICAL
SF36-PF												

For peer review only - http://bmjopen.bmj.com/site/about/guidelines.xhtml

Quality assessment							№ of patients		Effect		Quality	Importance
№ of studies	Study design	Risk of bias	Inconsistency	Indirectness	Imprecision	Other considerations	Early rehabiritation	Control	Relative (95% CI)	Absolute (95% CI)		
2	randomised trials	serious [a]	not serious	not serious	serious [b]	none	93	98	-	SMD **2.41 higher** (0.75 lower to 5.58 higher)	⨁⨁◯◯ LOW	CRITICAL

CI: Confidence interval; **SMD:** Standardized mean difference; **OR:** Odds ratio

a. Most of the studies are classified as unclear or high risk of bias (performance bias and attrition bias)

b. Confidence intervals for absolute effects are wide and include no effect and important harm and/or small sample size (The number of events is <300)

c. Substantial heterogeneity present

GRADE Working Group grades of evidence High quality: further research is very unlikely to change our confidence in the estimate of effect. Moderate quality: further research is likely to have an important impact on our confidence in the estimate of effect and may change the estimate. Low quality: further research is very likely to have an important impact on our confidence in the estimate of effect and is likely to change the estimate. Very low quality: we are very uncertain about the estimate.

For peer review only

Primary outcomes (short-term outcomes)

(i) Physical-related outcomes

Incidence of ICU-AW

Two RCTs reported the incidence of ICU-AW (Figure 3). There was a significant reduction in the incidence of ICU-AW in the early rehabilitation group according to the random effects model (two trials, n = 154; OR: 0.42, 95% CI: 0.22–0.82, p = 0.01; I^2 = 0%).

The risk of bias was determined to be serious because all RCTs included a moderate risk of bias. Imprecision was also determined to be serious because of the small number of included patients as per the optimal information size (OIS) criteria. Therefore, the QoE was downgraded from high to low because of these serious risks.

MRC

Three RCTs reported MRC scores (Figure 3). We observed a significant improvement in the early rehabilitation group [MRC (three trials, n = 196; SMD: 0.38, 95% CI: 0.10–0.66, p = 0.009; I^2 = 0%)].

(ii) Cognitive-related outcomes

Delirium-free days

Two RCTs reported the incidence of delirium-free days (Figure 3). There was no significant increase in this parameter in the early rehabilitation group according to a random effects model (two trials, n = 326; SMD: -0.02, 95% CI -0.23–0.20, p = 0.62; I^2 = 0%). The risk of bias was determined to be serious because all RCTs included a moderate risk of bias, and imprecision was also determined to be serious because of the small number of included patients as per OIS criteria. These serious risks

For peer review only - http://bmjopen.bmj.com/site/about/guidelines.xhtml

led to the downgrading of the QoE from high to low.

(iii) Mental health-related outcomes

Incidence of HAS/HADS

Two RCTs reported the incidence of HAS/HADS (Figure 3). These groups did not differ significantly according to a random effects model (two trials, n = 92; OR: 0.79, 95% CI: 0.29–2.12, p = 0.64; I^2 = 0%). The risk of bias was determined to be serious because all RCTs included a moderate risk of bias, and imprecision was also determined to be serious because of the small number of included patients. These serious risks led to the downgrading of the QoE from high to low.

Secondary outcomes (long-term outcomes)

EQ5D

Two RCTs reported the EQ5D scores (Figure 4). No significant difference was observed between the two groups according to a random effects model (two trials, n = 63; SMD**:** 0.11, 95% CI: -0.86–1.09, p = 0.82; I^2 = 72%). The risk of bias was deemed serious because all RCTs demonstrated a moderate risk of bias, and inconsistency was deemed serious because of severe heterogeneity (p = 0.06, I^2 = 72%). Imprecision was also considered serious because of the small number of included patients. These serious risks led to the downgrading of the QoE from high to very low.

SF-36 PF

Two RCTs reported SF-36 PF scale scores (Figure 4). However, these scores were evaluated several months later. Moreover, the timing of SF-36 PF evaluation varied greatly among studies (i.e., at hospital discharge and 2, 4, and 6 months after enrolment [32], or at 6 months after hospital discharge

For peer review only - http://bmjopen.bmj.com/site/about/guidelines.xhtml

[29]). We observed a significant improvement in the early rehabilitation group [SF-36 PF (two trials, n = 191; SMD: 2.41, 95% CI: -0.75–5.58, p = 0.14; I^2 = 98%)]. However, the different timings of SF-36 PF scale evaluation among the various studies led to significant heterogeneity. Imprecision was also considered serious because of the small number of included patients as per the OIS criteria. Therefore, these serious risks led to the downgrading of the QoE from high to very low.

For peer review only

For peer review only - http://bmjopen.bmj.com/site/about/guidelines.xhtml

DISCUSSION

Summary of the main results

This meta-analysis revealed that early rehabilitation significantly improved short-term physical function in critically ill patients, as assessed by the MRC scoring system and the incidence of ICU-AW. However, it did not significantly improve the patients' cognitive and mental status-related outcomes. These results suggest that early rehabilitation has a limited effect on the prevention of PICS in critically ill patients, and only reflect improvements in short-term physical function.

Strengths of the review

To our knowledge, the present work is the first meta-analysis of comprehensive PICS based on RCTs in which the study intervention populations were limited to early rehabilitation. Additionally, we used the GRADE approach in the review process, which allowed us to make judgments about the quality of evidence and strength of recommendations in a systematic and transparent manner. Thus, the present meta-analysis allowed us determine whether critical care physicians should initiate early rehabilitation for critically ill patients, and provided new evidence about the effectiveness of physiotherapy interventions aimed at early mobilisation [33].

Agreements and disagreements with other studies or reviews

Four systematic reviews have addressed the effect of rehabilitation [33-35] or early rehabilitation [36] during an ICU stay on functional status. Through a meta-analysis, Kayambu et al. found that physical therapy in the ICU may improve muscle strength, physical function, quality of life, ventilator-free days, ICU stay, and hospital stay; however, the authors included one RCT involving patients who underwent laparotomy (among 10 total RCTs), and another study initiated physical

For peer review only - http://bmjopen.bmj.com/site/about/guidelines.xhtml

therapy 30 days after bed rest [34]. Therefore, the results cannot be directly applied to our clinical practice because of the heterogeneity of the study population. The present meta-analysis showed that early rehabilitation did not improve the patients' quality of life. The inconsistency of these results might be attributable to differences in the methods used to assess quality of life. Kayambu et al. used the Medical Outcomes Study 36-Item Short Form survey score, whereas the present review included used the EQ5D.

Castro-Avila et al. conducted a systematic review and meta-analysis of the effect of early rehabilitation during an ICU stay on functional status [34], and included the study reported by Denehy et al [37]. However, we did not include this study in our systematic review, as it implemented early active rehabilitation in the control group. This implementation was the main reason underlying the authors' conclusion that early rehabilitation was not associated with improvements in the walking distance and incidence of ICU-AW. Furthermore, Tipping et al. reported a systematic review regarding the effects of active mobilisation and rehabilitation in the ICU on mortality and function [35], and included a study by Moss et al [38]. However, that study did not initiate rehabilitation during the early phase and therefore was also not included in the current systematic review.

Clinical implementation

The initiation of early rehabilitation improved short-term outcomes, such as the physical function score and the incidence of ICU-AW in the present systematic review; however, it did not improve. The tendency to favour early rehabilitation during mechanical ventilation suggests that early rehabilitation could potentially improve short-term survival and maintain and increase muscle strength.

However, the initiation of rehabilitation during acute phase may inflict tremendous physical stress and exhaustion upon patients in the ICU, thus increasing ICU mortality [39]. Although several

For peer review only - http://bmjopen.bmj.com/site/about/guidelines.xhtml

studies included in the present meta-analysis described the safety of early physiotherapy in the ICU [27 29-32 40], only two outcomes related to the safety of early rehabilitation, the blood lactate level [29] and early physiotherapy termination rate [31], were compared between two groups. Therefore, the study conclusions should be approached with caution.

Limitations of the review

The current **meta-analysis** had several limitations. First, the analysis of each outcome included only a small number of patients. Although Schweickert et al. reported data concerning delirium, we could not extract cognitive-related outcomes because no data about delirium-free days were not addressed directly [30]. Second, the studies included in the current systematic review did not measure the domains of PICS beyond hospital discharge. Third, two of the included studies were pilot/feasibility trials and not powered to identify differences in physical or cognitive outcomes [27 31]. However, it was reasonable to initiate RCTs in the context of feasibility studies because no large multicentre trials of the effects of early mobilisation in the ICU had been published before the study conducted by Brummel et al., and little evidence to support the feasibility of individual patient randomisation across multiple sites using a complex intervention (i.e. early mobilisation) had been reported before the study conducted by Hodgson et al. Moreover, these two RCTs demonstrated the same level of risk as other RCTs, according to risk of bias summary conducted in this meta-analysis (Figure 2). Fourth, we could not confirm the exact first day of rehabilitation initiation in the three studies, although 'early rehabilitation' was described in the text and we contacted the authors to acquire additional information [28 40]. Fifth, we could not find any data regarding direct cognitive assessment in the selected papers. Although cognitive impairment at hospital discharge may not predict long-term cognitive impairment [41], Pandharipande et al. reported that a longer duration of delirium upon admission was associated with worse global cognition and executive function scores in the

For peer review only - http://bmjopen.bmj.com/site/about/guidelines.xhtml

surgical ICU at 3 and 12 months [42]. Therefore, we believe that the assessment of delirium in the ICU and hospital is a very important predictor of long-term cognitive impairment post-discharge. Sixth, as this meta-analysis included studies that had implemented various 'early rehabilitation' protocols, further analyses should include large trials with strict and comparable rehabilitation algorithms adjusted for the initiation time, type, and intensity. Despite these several limitations, our meta-analysis clarifies the effectiveness of early rehabilitation for the prevention of PICS in survivors of critical illness.

CONCLUSION

Early rehabilitation has a limited effect on the prevention of PICS, although it led to significant improvements in short-term physical-related outcomes, including MRC scores and the incidence of ICU-AW. However, early rehabilitation had no significant effect on cognitive function and mental health-related outcomes or mortality in critically ill patients. Additional large-scale, rigorous RCTs are needed to confirm our results.

For peer review only

For peer review only - http://bmjopen.bmj.com/site/about/guidelines.xhtml

Contributors

YK, RF, TH, JH, TT, and SI conceived the idea for this systematic review. YK, RF, TH, JH, TT, and SI developed the methodology for the systematic review, and KY and ON supervised the methodological process. The manuscript was drafted by YK and SI. RF, TH, JH, TT, and KY and revised by ON. All authors critically reviewed and approved the final manuscript.

Funding

No funding support was received for this systematic review meta-analysis.

Competing interests

None declared.

Provenance and peer review

This work was not commissioned and was externally peer reviewed.

Data sharing statement Requests for additional details regarding the review and meta-analysis protocol may be made by contacting the corresponding author, Dr Shigeaki Inoue (caf55000@gmail.com).

For peer review only

For peer review only - http://bmjopen.bmj.com/site/about/guidelines.xhtml

FIGURE LEGENDS

Figure 1

Flow chart of the process of study identification and inclusion

Figure 2

Risk of bias summary

Figure 3

The effect of early rehabilitation on short-term outcomes in post-intensive care syndrome (PICS) in intensive care unit (ICU) patients.1) Physical-related outcomes **A.** Incidence of ICU-acquired weakness (AW). **B.** Medical Research Council sum score. 2) Cognitive-related outcomes, characterised by delirium-free days. 3) Mental status-related outcomes, characterised by the Hospital Anxiety and Depression Scale (HAS/HADS) score. CI, confidence interval

Figure 4

The effect of early rehabilitation on long-term outcomes in post-intensive care syndrome (PICS) in intensive care unit (ICU) patients. The effect of early rehabilitation on health-related quality of life (QOL) scores and in ICU patients. 1) Health-related quality of life (QOL) scores calculated from the EuroQol 5 Dimensions (EQ5D). 2) Medical Outcomes Study 36-Item Short Form Health Survey Physical Function scale (SF-36 PF).

For peer review only

REFERENCES

1. Bemis-Dougherty AR, Smith JM. What follows survival of critical illness? Physical therapists' management of patients with post-intensive care syndrome. *Phys Ther* 2013;93(2):179-85. doi: 10.2522/ptj.20110429
2. Iwashyna TJ, Ely EW, Smith DM, et al. Long-term cognitive impairment and functional disability among survivors of severe sepsis. *JAMA : the journal of the American Medical Association* 2010;304(16):1787-94. doi: 10.1001/jama.2010.1553
3. Desai SV, Law TJ, Needham DM. Long-term complications of critical care. *Crit Care Med* 2011;39(2):371-9. doi: 10.1097/CCM.0b013e3181fd66e5
4. Needham DM, Davidson J, Cohen H, et al. Improving long-term outcomes after discharge from intensive care unit: report from a stakeholders' conference. *Crit Care Med* 2012;40(2):502-9. doi: 10.1097/CCM.0b013e318232da75
5. Elliott D, Davidson JE, Harvey MA, et al. Exploring the scope of post-intensive care syndrome therapy and care: engagement of non-critical care providers and survivors in a second stakeholders meeting. *Crit Care Med* 2014;42(12):2518-26. doi: 10.1097/CCM.0000000000000525
6. Gielen S, Adams V, Möbius-Winkler S, et al. Anti-inflammatory effects of exercise training in the skeletal muscle of patients with chronic heart failure. *J Am Coll Cardiol* 2003;42(5):861-68. doi: 10.1016/S0735-1097(03)00848-9
7. Nuhr MJ, Pette D, Berger R, et al. Beneficial effects of chronic low-frequency stimulation of thigh muscles in patients with advanced chronic heart failure. *Eur Heart J* 2004;25(2):136-43. doi: 10.1016/j.ehj.2003.09.027
8. Vivodtzev I, Pépin JL, Vottero G, et al. Improvement in quadriceps strenght and dyspnea in daily tasks after 1 month of electrical stimulation in severely deconditioned and malnourished COPD. *Chest* 2006;129(6):1540-48. doi: 10.1378/chest.129.6.1540
9. Laaksonen DE, Atalay M, Niskanen L, et al. Increased resting and exercise-induced oxidative stress in young IDDM men. *Diabetes care* 1996;19(6):569-74.
10. Mercken EM, Hageman GJ, Schols AMWJ, et al. Rehabilitation decreases exercise-induced oxidative stress in chronic obstructive pulmonary disease. *American Journal of Respiratory and Critical Care Medicine* 2005;172(8):994-1001. doi: 10.1164/rccm.200411-1580OC
11. Nishiyama Y, Ikeda H, Haramaki N, et al. Oxidative stress is related to exercise intolerance in patients with heart failure. *Am Heart J* 1998;135(1):115-20. doi: 10.1016/S0002-8703(98)70351-5
12. Vincent HK, Morgan JW, Vincent KR. Obesity Exacerbates Oxidative Stress Levels after Acute Exercise. *Med Sci Sports Exerc* 2004;36(5):772-79.

For peer review only - http://bmjopen.bmj.com/site/about/guidelines.xhtml

13. Moher D, Booth A, Stewart L. How to reduce unnecessary duplication: use PROSPERO. *BJOG : an international journal of obstetrics and gynaecology* 2014;121(7):784-6. doi: 10.1111/1471-0528.12657

14. Moher D, Shamseer L, Clarke M, et al. Preferred reporting items for systematic review and meta-analysis protocols (PRISMA-P) 2015 statement. *Systematic reviews* 2015;4:1. doi: 10.1186/2046-4053-4-1

15. Shamseer L, Moher D, Clarke M, et al. Preferred reporting items for systematic review and meta-analysis protocols (PRISMA-P) 2015: elaboration and explanation. *BMJ* 2015;349:g7647. doi: 10.1136/bmj.g7647

16. Liberati A, Altman DG, Tetzlaff J, et al. The PRISMA statement for reporting systematic reviews and meta-analyses of studies that evaluate health care interventions: explanation and elaboration. *J Clin Epidemiol* 2009;62(10):e1-34. doi: 10.1016/j.jclinepi.2009.06.006

17. Moher D, Liberati A, Tetzlaff J, et al. Preferred reporting items for systematic reviews and meta-analyses: the PRISMA statement. *PLoS medicine* 2009;6(7):e1000097. doi: 10.1371/journal.pmed.1000097

18. Kondo Y, Fuke R, Hifumi T, et al. Early rehabilitation for the prevention of postintensive care syndrome in critically ill patients: a study protocol for a systematic review and meta-analysis. *BMJ open* 2017;7(3):e013828. doi: 10.1136/bmjopen-2016-013828

19. Paternostro-Sluga T, Grim-Stieger M, Posch M, et al. Reliability and validity of the Medical Research Council (MRC) scale and a modified scale for testing muscle strength in patients with radial palsy. *Journal of rehabilitation medicine* 2008;40(8):665-71. doi: 10.2340/16501977-0235

20. Yang ZZ, Grote DM, Xiu B, et al. TGF-beta upregulates CD70 expression and induces exhaustion of effector memory T cells in B-cell non-Hodgkin's lymphoma. *Leukemia : official journal of the Leukemia Society of America, Leukemia Research Fund, UK* 2014;28(9):1872-84. doi: 10.1038/leu.2014.84

21. Mozzi A, Meregaglia M, Lazzaro C, et al. A comparison of EuroQol 5-Dimension health-related utilities using Italian, UK, and US preference weights in a patient sample. *Clinicoecon Outcomes Res* 2016;8:267-74. doi: 10.2147/CEOR.S98226

22. Obradovic M, Lal A, Liedgens H. Validity and responsiveness of EuroQol-5 dimension (EQ-5D) versus Short Form-6 dimension (SF-6D) questionnaire in chronic pain. *Health Qual Life Outcomes* 2013;11:110. doi: 10.1186/1477-7525-11-110

23. Yang X, Fan D, Xia Q, et al. The health-related quality of life of ankylosing spondylitis patients assessed by SF-36: a systematic review and meta-analysis. *Qual Life Res* 2016;25(11):2711-23. doi: 10.1007/s11136-016-1345-z

For peer review only

For peer review only - http://bmjopen.bmj.com/site/about/guidelines.xhtml

24. JPT H. Chapter 8: Assessing risk of bias in included studies. 2011

25. Duan EH, Oczkowski SJ, Belley-Cote E, et al. beta-Blockers in sepsis: protocol for a systematic review and meta-analysis of randomised control trials. *BMJ open* 2016;6(6):e012466. doi: 10.1136/bmjopen-2016-012466

26. Klotz R, Klaiber U, Grummich K, et al. Percutaneous versus surgical strategy for tracheostomy: protocol for a systematic review and meta-analysis of perioperative and postoperative complications. *Systematic reviews* 2015;4:105. doi: 10.1186/s13643-015-0092-5

27. Brummel NE, Girard TD, Ely EW, et al. Feasibility and safety of early combined cognitive and physical therapy for critically ill medical and surgical patients: the Activity and Cognitive Therapy in ICU (ACT-ICU) trial. *Intensive Care Med* 2014;40(3):370-9. doi: 10.1007/s00134-013-3136-0

28. Jones C, Eddleston J, McCairn A, et al. Improving rehabilitation after critical illness through outpatient physiotherapy classes and essential amino acid supplement: A randomized controlled trial. *Journal of critical care* 2015 doi: 10.1016/j.jcrc.2015.05.002

29. Kayambu G, Boots R, Paratz J. Early physical rehabilitation in intensive care patients with sepsis syndromes: a pilot randomised controlled trial. *Intensive Care Med* 2015;41(5):865-74. doi: 10.1007/s00134-015-3763-8

30. Schweickert WD, Pohlman MC, Pohlman AS, et al. Early physical and occupational therapy in mechanically ventilated, critically ill patients: a randomised controlled trial. *Lancet* 2009;373(9678):1874-82. doi: 10.1016/S0140-6736(09)60658-9

31. Hodgson CL, Bailey M, Bellomo R, et al. A Binational Multicenter Pilot Feasibility Randomized Controlled Trial of Early Goal-Directed Mobilization in the ICU. *Crit Care Med* 2016;44(6):1145-52. doi: 10.1097/CCM.0000000000001643

32. Morris PE, Berry MJ, Files DC, et al. Standardized Rehabilitation and Hospital Length of Stay Among Patients With Acute Respiratory Failure: A Randomized Clinical Trial. *JAMA : the journal of the American Medical Association* 2016;315(24):2694-702. doi: 10.1001/jama.2016.7201

33. Stiller K. Physiotherapy in intensive care: an updated systematic review. *Chest* 2013;144(3):825-47. doi: 10.1378/chest.12-2930

34. Kayambu G, Boots R, Paratz J. Physical therapy for the critically ill in the ICU: a systematic review and meta-analysis. *Crit Care Med* 2013;41(6):1543-54. doi: 10.1097/CCM.0b013e31827ca637

35. Tipping CJ, Harrold M, Holland A, et al. The effects of active mobilisation and rehabilitation in ICU on mortality and function: a systematic review. *Intensive Care Med* 2016 doi: 10.1007/s00134-016-4612-0

36. Castro-Avila AC, Seron P, Fan E, et al. Effect of Early Rehabilitation during Intensive Care Unit Stay

For peer review only

For peer review only - http://bmjopen.bmj.com/site/about/guidelines.xhtml

on Functional Status: Systematic Review and Meta-Analysis. *PloS one* 2015;10(7):e0130722. doi: 10.1371/journal.pone.0130722

37. Denehy L, Skinner EH, Edbrooke L, et al. Exercise rehabilitation for patients with critical illness: a randomized controlled trial with 12 months of follow-up. *Crit Care* 2013;17(4):R156. doi: 10.1186/cc12835 [published Online First: 2013/07/26]
38. Moss M, Nordon-Craft A, Malone D, et al. A Randomized Trial of an Intensive Physical Therapy Program for Patients with Acute Respiratory Failure. *Am J Respir Crit Care Med* 2016;193(10):1101-10. doi: 10.1164/rccm.201505-1039OC
39. Greening NJ, Williams JE, Hussain SF, et al. An early rehabilitation intervention to enhance recovery during hospital admission for an exacerbation of chronic respiratory disease: randomised controlled trial. *BMJ* 2014;349:g4315. doi: 10.1136/bmj.g4315
40. Cameron JI, Chu LM, Matte A, et al. One-Year Outcomes in Caregivers of Critically Ill Patients. *The New England journal of medicine* 2016;374(19):1831-41. doi: 10.1056/NEJMoa1511160
41. Woon FL, Dunn CB, Hopkins RO. Predicting cognitive sequelae in survivors of critical illness with cognitive screening tests. *Am J Respir Crit Care Med* 2012;186(4):333-40. doi: 10.1164/rccm.201112-2261OC
42. Pandharipande PP, Girard TD, Jackson JC, et al. Long-term cognitive impairment after critical illness. *The New England journal of medicine* 2013;369(14):1306-16. doi: 10.1056/NEJMoa1301372

For peer review only

For peer review only - http://bmjopen.bmj.com/site/about/guidelines.xhtml

Figure 1

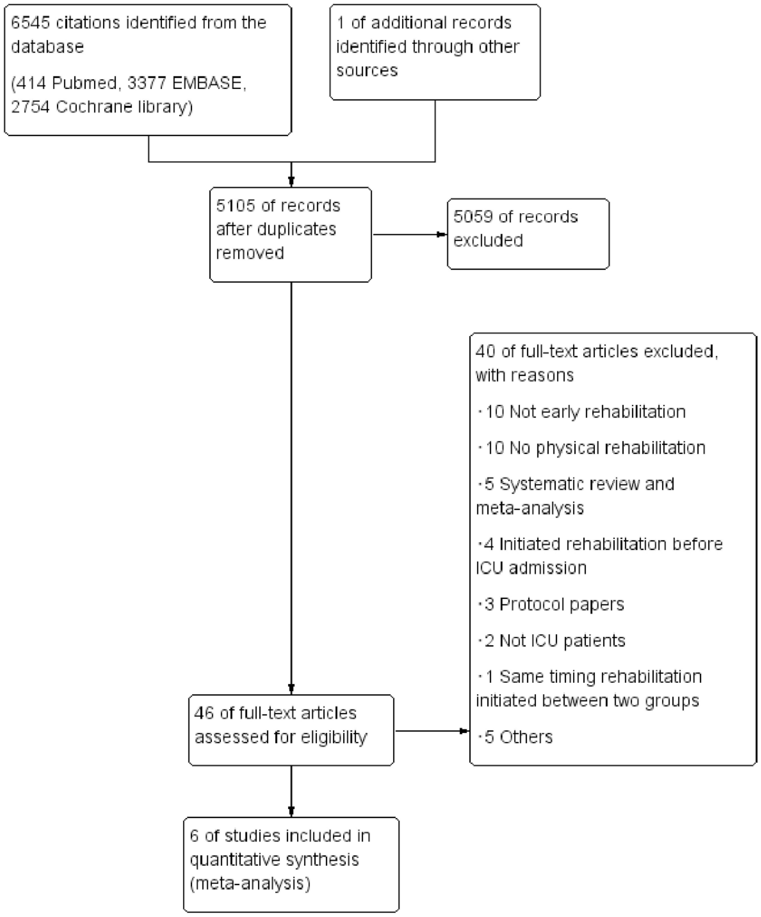

Figure 1

254x338mm (300 x 300 DPI)

Figure 2

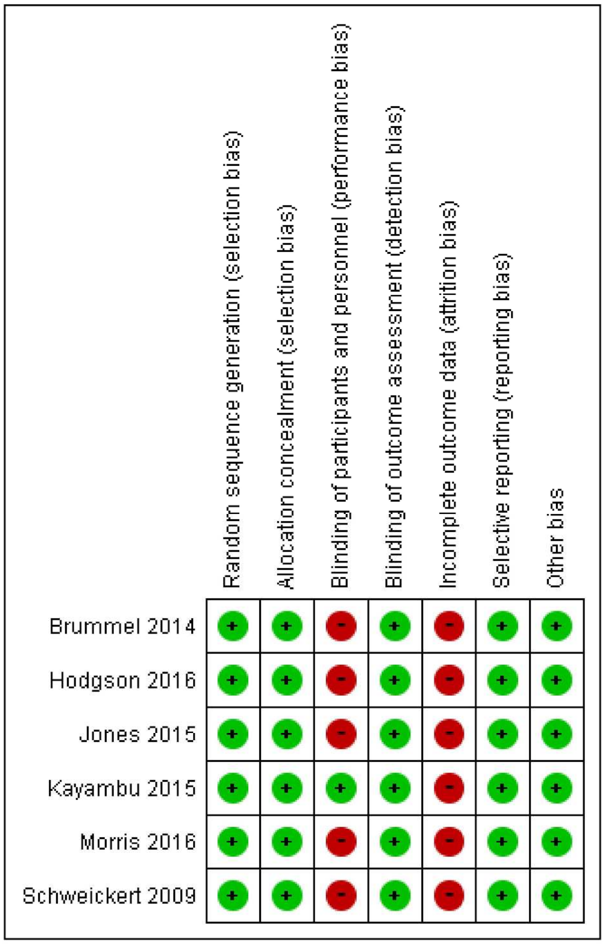

Figure 2

254x338mm (300 x 300 DPI)

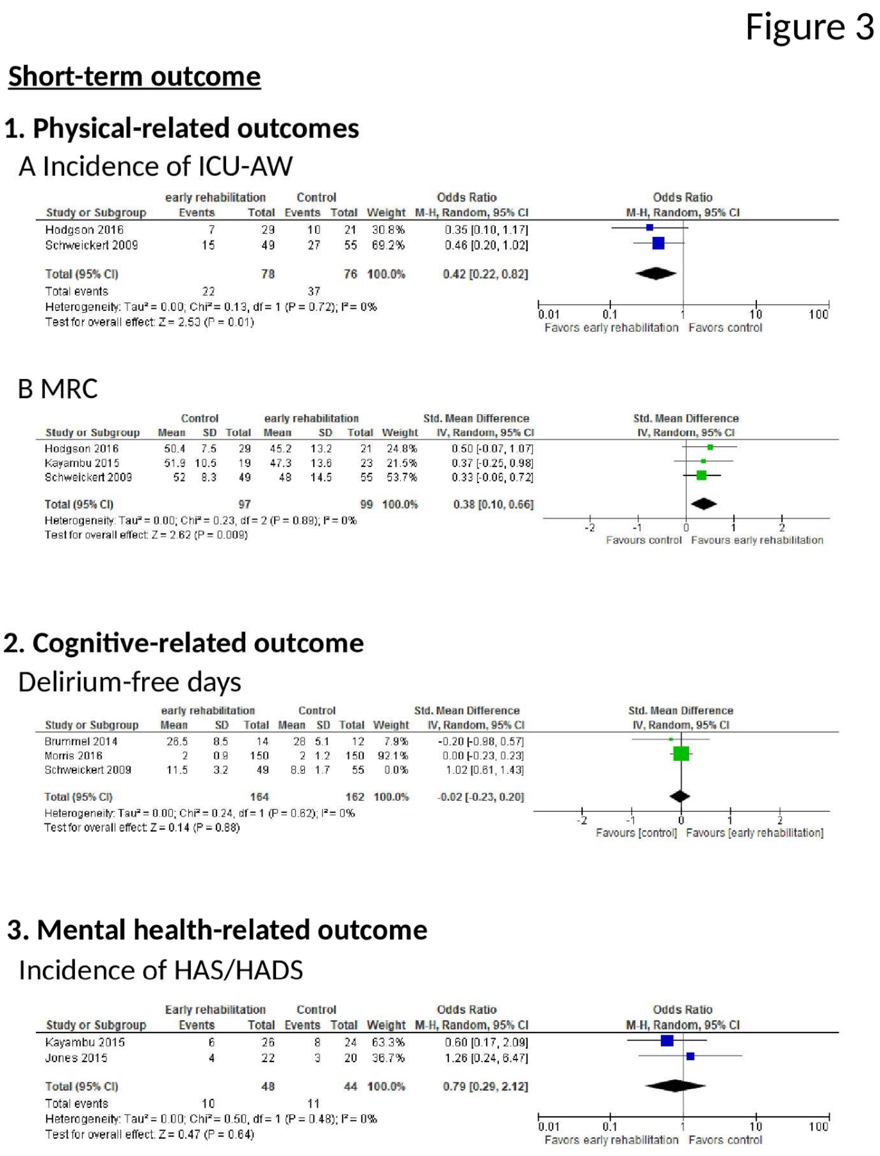

Figure 3

254x338mm (300 x 300 DPI)

Figure 4

Long-term outcome

1 Health-related QOL scores

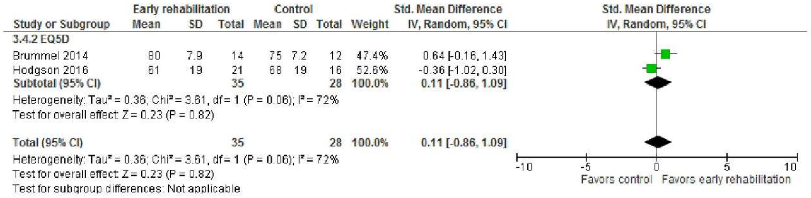

2 SF-36PF

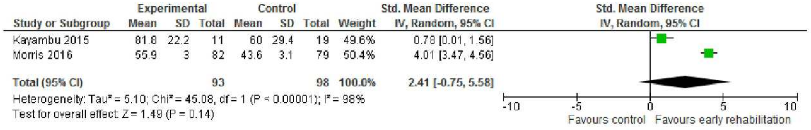

Figure 4

254x338mm (300 x 300 DPI)

Supplementary file 1.

The full search strategies for Medline (via PubMed), EMBASE and the Cochrane Central Register of clinical trials.

#1 ("critical ill" or "critical illness" or "critical care" or "intensive care" or "mechanical ventilation" or "mechanical ventilated" or "postoperative care")

#2 (rehabilitation or "physical therapy" or physiotherapy or exercise or mobilization or "mobility intervention" or "muscle training")

#3 ("Activities of Daily Living" or "Quality of Life" or "post-intensive care syndrome" or "motor function" or "Physical Functioning" or "functional status" or "physical function" or "ventilator days" or "quality of life" or (walking or walk) or muscle or polyneuromyopathy' or "length of stay" or "length of ICU stay" or "length of hospital stay" or "intubation period" or "duration of mechanical ventilation" or re-admission or "functional outcome" or "ICU-acquired weakness" or "ICU-acquired paresis" or ICUAW or "ICU-AW" or "intensive care unit acquired weakness" or "critical illness polyneuropathy" or "critical illness myopathy" or "critical illness neuromyopathy" or "acute quadriplegic myopathy" or "thick filament myopathy" or "acute necrotizing myopathy of intensive care" or "acute corticosteroid myopathy" or "critical illness neuromuscula syndromes" or "Tower test" or "Timed Up and Go Test" or "dysexecutive questionnaire" or FAQ or "EQ-5D VAS" or 6MWD or "6-min walking distance" or "Quadriceps force, and self-perceived functional status" or "SF-36 PF" or MRC or "Medical Research Council" or "AQoL utility" or "EQ-5D" or PFIT or "physical functional ICU test" or "Hospital Anxiety and Depression Scale" or "Hand-grip strength")

#4 #1 AND #2 AND #3

Article search was performed at 7 July 2016 and a total of 5104 articles are scanned.

For peer review only

Supplementary file 2

List of excluded studies

40 excluded studies with reasons

10 No early rehabilitation [1-10]

10 No physical rehabilitation [11-20]

5 Systematic review and meta-analysis [21-25]

4 Initiated rehabilitation before ICU admission [26-29]

3 Protocol paper [30-32]

2 Not ICU patients [33 34]

1 Same timing rehabilitation initiated between two groups [35]

5 Others [36-40]

For peer review only

References

1. Stein R, Maia CP, Silveira AD, et al. Inspiratory muscle strength as a determinant of functional capacity early after coronary artery bypass graft surgery. *Archives of physical medicine and rehabilitation* 2009;90(10):1685-91. doi: 10.1016/j.apmr.2009.05.010
2. Patman S, Sanderson D, Blackmore M. Physiotherapy following cardiac surgery: is it necessary during the intubation period? *The Australian journal of physiotherapy* 2001;47(1):7-16.
3. Porta R, Vitacca M, Gile LS, et al. Supported arm training in patients recently weaned from mechanical ventilation. *Chest* 2005;128(4):2511-20. doi: 10.1378/chest.128.4.2511
4. Jackson JC, Ely EW, Morey MC, et al. Cognitive and physical rehabilitation of intensive care unit survivors: results of the RETURN randomized controlled pilot investigation. *Crit Care Med* 2012;40(4):1088-97. doi: 10.1097/CCM.0b013e3182373115
5. Patman S, Jenkins S, Stiller K. Physiotherapy does not prevent, or hasten recovery from, ventilator-associated pneumonia in patients with acquired brain injury. *Intensive Care Med* 2009;35(2):258-65. doi: 10.1007/s00134-008-1278-2
6. Chen S, Su CL, Wu YT, et al. Physical training is beneficial to functional status and survival in patients

with prolonged mechanical ventilation. *Journal of the Formosan Medical Association = Taiwan yi zhi* 2011;110(9):572-9. doi: 10.1016/j.jfma.2011.07.008

7. Walsh TS, Salisbury LG, Merriweather JL, et al. Increased Hospital-Based Physical Rehabilitation and Information Provision After Intensive Care Unit Discharge: The RECOVER Randomized Clinical Trial. *JAMA internal medicine* 2015;175(6):901-10. doi: 10.1001/jamainternmed.2015.0822
8. Chiang LL, Wang LY, Wu CP, et al. Effects of physical training on functional status in patients with prolonged mechanical ventilation. *Phys Ther* 2006;86(9):1271-81. doi: 10.2522/ptj.20050036
9. Chen YH, Lin HL, Hsiao HF, et al. Effects of exercise training on pulmonary mechanics and functional status in patients with prolonged mechanical ventilation. *Respir Care* 2012;57(5):727-34. doi: 10.4187/respcare.01341
10. Burtin C, Clerckx B, Robbeets C, et al. Early exercise in critically ill patients enhances short-term functional recovery. *Crit Care Med* 2009;37(9):2499-505. doi: 10.1097/CCM.0b013e3181a38937
11. Jackson JC, Girard TD, Gordon SM, et al. Long-term cognitive and psychological outcomes in the awakening and breathing controlled trial. *Am J Respir Crit Care Med* 2010;182(2):183-91. doi: 10.1164/rccm.200903-0442OC
12. Nava S. Rehabilitation of patients admitted to a respiratory intensive care unit. *Archives of physical medicine and rehabilitation* 1998;79(7):849-54.
13. Mackay MR, Ellis E, Johnston C. Randomised clinical trial of physiotherapy after open abdominal surgery in high risk patients. *The Australian journal of physiotherapy* 2005;51(3):151-9.
14. Stiller K, Montarello J, Wallace M, et al. Efficacy of breathing and coughing exercises in the prevention of pulmonary complications after coronary artery surgery. *Chest* 1994;105(3):741-7.
15. Caruso P, Denari SD, Ruiz SA, et al. Inspiratory muscle training is ineffective in mechanically ventilated critically ill patients. *Clinics (Sao Paulo)* 2005;60(6):479-84. doi: /S1807-59322005000600009
16. Albert NM, Gillinov AM, Lytle BW, et al. A randomized trial of massage therapy after heart surgery. *Heart Lung* 2009;38(6):480-90. doi: 10.1016/j.hrtlng.2009.03.001
17. Pattanshetty RB, Gaude GS. Effect of multimodality chest physiotherapy on the rate of recovery and prevention of complications in patients with mechanical ventilation: a prospective study in medical and surgical intensive care units. *Indian J Med Sci* 2011;65(5):175-85. doi: 10.4103/0019-5359.106608
18. Templeton M, Palazzo MG. Chest physiotherapy prolongs duration of ventilation in the critically ill ventilated for more than 48 hours. *Intensive Care Med* 2007;33(11):1938-45. doi: 10.1007/s00134-007-0762-4
19. Cader SA, Vale RG, Castro JC, et al. Inspiratory muscle training improves maximal inspiratory pressure and may assist weaning in older intubated patients: a randomised trial. *Journal of physiotherapy* 2010;56(3):171-7.

For peer review only

20. Pattanshetty RB, Gaude GS. Effect of multimodality chest physiotherapy in prevention of ventilator-associated pneumonia: A randomized clinical trial. *Indian journal of critical care medicine : peer-reviewed, official publication of Indian Society of Critical Care Medicine* 2010;14(2):70-6. doi: 10.4103/0972-5229.68218

21. Patel BK, Pohlman AS, Hall JB, et al. Impact of early mobilization on glycemic control and ICU-acquired weakness in critically ill patients who are mechanically ventilated. *Chest* 2014;146(3):583-9. doi: 10.1378/chest.13-2046

22. Connolly B, Salisbury L, O'Neill B, et al. Exercise rehabilitation following intensive care unit discharge for recovery from critical illness. *Cochrane Database Syst Rev* 2015(6):CD008632. doi: 10.1002/14651858.CD008632.pub2

23. Mehrholz J, Pohl M, Kugler J, et al. Physical rehabilitation for critical illness myopathy and neuropathy. *Cochrane Database Syst Rev* 2015(3):CD010942. doi: 10.1002/14651858.CD010942.pub2

24. Elkins M, Dentice R. Inspiratory muscle training facilitates weaning from mechanical ventilation among patients in the intensive care unit: a systematic review. *Journal of physiotherapy* 2015;61(3):125-34. doi: 10.1016/j.jphys.2015.05.016

25. Castro-Avila AC, Seron P, Fan E, et al. Effect of Early Rehabilitation during Intensive Care Unit Stay on Functional Status: Systematic Review and Meta-Analysis. *PloS one* 2015;10(7):e0130722. doi: 10.1371/journal.pone.0130722

26. Herdy AH, Marcchi PL, Vila A, et al. Pre- and postoperative cardiopulmonary rehabilitation in hospitalized patients undergoing coronary artery bypass surgery: a randomized controlled trial. *Am J Phys Med Rehabil* 2008;87(9):714-9. doi: 10.1097/PHM.0b013e3181839152

27. Arthur HM, Daniels C, McKelvie R, et al. Effect of a preoperative intervention on preoperative and postoperative outcomes in low-risk patients awaiting elective coronary artery bypass graft surgery. A randomized, controlled trial. *Annals of internal medicine* 2000;133(4):253-62.

28. Fagevik Olsen M, Hahn I, Nordgren S, et al. Randomized controlled trial of prophylactic chest physiotherapy in major abdominal surgery. *Br J Surg* 1997;84(11):1535-8.

29. Weiner P, Zeidan F, Zamir D, et al. Prophylactic inspiratory muscle training in patients undergoing coronary artery bypass graft. *World journal of surgery* 1998;22(5):427-31.

30. Kayambu G, Boots RJ, Paratz JD. Early rehabilitation in sepsis: a prospective randomised controlled trial investigating functional and physiological outcomes The i-PERFORM Trial (Protocol Article). *BMC anesthesiology* 2011;11:21. doi: 10.1186/1471-2253-11-21

31. Thomas K, Wright SE, Watson G, et al. Extra Physiotherapy in Critical Care (EPICC) Trial Protocol: a randomised controlled trial of intensive versus standard physical rehabilitation therapy in the critically ill. *BMJ open* 2015;5(5):e008035. doi: 10.1136/bmjopen-2015-008035

32. Brummel NE, Jackson JC, Girard TD, et al. A combined early cognitive and physical rehabilitation

For peer review only

program for people who are critically ill: the activity and cognitive therapy in the intensive care unit (ACT-ICU) trial. *Phys Ther* 2012;92(12):1580-92. doi: 10.2522/ptj.20110414

33. Reeve JC, Nicol K, Stiller K, et al. Does physiotherapy reduce the incidence of postoperative pulmonary complications following pulmonary resection via open thoracotomy? A preliminary randomised single-blind clinical trial. *Eur J Cardiothorac Surg* 2010;37(5):1158-66. doi: 10.1016/j.ejcts.2009.12.011
34. Lee SM, Kang SB, Jang JH, et al. Early rehabilitation versus conventional care after laparoscopic rectal surgery: a prospective, randomized, controlled trial. *Surgical endoscopy* 2013;27(10):3902-9. doi: 10.1007/s00464-013-3006-4
35. Hirschhorn AD, Richards DA, Mungovan SF, et al. Does the mode of exercise influence recovery of functional capacity in the early postoperative period after coronary artery bypass graft surgery? A randomized controlled trial. *Interactive cardiovascular and thoracic surgery* 2012;15(6):995-1003. doi: 10.1093/icvts/ivs403
36. Yosef-Brauner O, Adi N, Ben Shahar T, et al. Effect of physical therapy on muscle strength, respiratory muscles and functional parameters in patients with intensive care unit-acquired weakness. *The clinical respiratory journal* 2015;9(1):1-6. doi: 10.1111/crj.12091
37. Denehy L, Skinner EH, Edbrooke L, et al. Exercise rehabilitation for patients with critical illness: a randomized controlled trial with 12 months of follow-up. *Crit Care* 2013;17(4):R156. doi: 10.1186/cc12835
38. Melchers P, Maluck A, Suhr L, et al. An early onset rehabilitation program for children and adolescents after traumatic brain injury (TBI): Methods and first results. *Restorative neurology and neuroscience* 1999;14(2):153-60.
39. Dantas CM, Silva PF, Siqueira FH, et al. Influence of early mobilization on respiratory and peripheral muscle strength in critically ill patients. *Revista brasileira de terapia intensiva* 2012;24(2):173-8.
40. Dong ZH, Yu BX, Sun YB, et al. Effects of early rehabilitation therapy on patients with mechanical ventilation. *World journal of emergency medicine* 2014;5(1):48-52. doi: 10.5847/wjem.j.1920-8642.2014.01.008

For peer review only

PRISMA 2009 Checklist

Section/topic	#	Checklist item	Reported on page #
**TITLE**			
Title	1	Identify the report as a systematic review, meta-analysis, or both.	1
**ABSTRACT**			
Structured summary	2	Provide a structured summary including, as applicable: background; objectives; data sources; study eligibility criteria, participants, and interventions; study appraisal and synthesis methods; results; limitations; conclusions and implications of key findings; systematic review registration number.	3
**INTRODUCTION**			
Rationale	3	Describe the rationale for the review in the context of what is already known.	5
Objectives	4	Provide an explicit statement of questions being addressed with reference to participants, interventions, comparisons, outcomes, and study design (PICOS).	5
**METHODS**			
Protocol and registration	5	Indicate if a review protocol exists, if and where it can be accessed (e.g., Web address), and, if available, provide registration information including registration number.	6
Eligibility criteria	6	Specify study characteristics (e.g., PICOS, length of follow-up) and report characteristics (e.g., years considered, language, publication status) used as criteria for eligibility, giving rationale.	6, 7
Information sources	7	Describe all information sources (e.g., databases with dates of coverage, contact with study authors to identify additional studies) in the search and date last searched.	6, 8
Search	8	Present full electronic search strategy for at least one database, including any limits used, such that it could be repeated.	Suppl 1
Study selection	9	State the process for selecting studies (i.e., screening, eligibility, included in systematic review, and, if applicable, included in the meta-analysis).	7, 8, 9
Data collection process	10	Describe method of data extraction from reports (e.g., piloted forms, independently, in duplicate) and any processes for obtaining and confirming data from investigators.	8
Data items	11	List and define all variables for which data were sought (e.g., PICOS, funding sources) and any assumptions and simplifications made.	-
Risk of bias in individual studies	12	Describe methods used for assessing risk of bias of individual studies (including specification of whether this was done at the study or outcome level), and how this information is to be used in any data synthesis.	7, 8
Summary measures	13	State the principal summary measures (e.g., risk ratio, difference in means).	8, 9
Synthesis of results	14	Describe the methods of handling data and combining results of studies, if done, including measures of consistency (e.g., I^2) for each meta-analysis.	8, 9

For peer review only - http://bmjopen.bmj.com/site/about/guidelines.xhtml

PRISMA 2009 Checklist

Section/topic	#	Checklist item	Reported on page #
Risk of bias across studies	15	Specify any assessment of risk of bias that may affect the cumulative evidence (e.g., publication bias, selective reporting within studies).	9
Additional analyses	16	Describe methods of additional analyses (e.g., sensitivity or subgroup analyses, meta-regression), if done, indicating which were pre-specified.	-
**RESULTS**			
Study selection	17	Give numbers of studies screened, assessed for eligibility, and included in the review, with reasons for exclusions at each stage, ideally with a flow diagram.	10, Figure 1
Study characteristics	18	For each study, present characteristics for which data were extracted (e.g., study size, PICOS, follow-up period) and provide the citations.	Table 1
Risk of bias within studies	19	Present data on risk of bias of each study and, if available, any outcome level assessment (see item 12).	Figure 2
Results of individual studies	20	For all outcomes considered (benefits or harms), present, for each study: (a) simple summary data for each intervention group (b) effect estimates and confidence intervals, ideally with a forest plot.	Figure 3, 4, 5
Synthesis of results	21	Present results of each meta-analysis done, including confidence intervals and measures of consistency.	11, 12, 13, Figure 3
Risk of bias across studies	22	Present results of any assessment of risk of bias across studies (see Item 15).	11, 12, 13, Figure 3
Additional analysis	23	Give results of additional analyses, if done (e.g., sensitivity or subgroup analyses, meta-regression [see Item 16]).	-
**DISCUSSION**			
Summary of evidence	24	Summarize the main findings including the strength of evidence for each main outcome; consider their relevance to key groups (e.g., healthcare providers, users, and policy makers).	14
Limitations	25	Discuss limitations at study and outcome level (e.g., risk of bias), and at review-level (e.g., incomplete retrieval of identified research, reporting bias).	16
Conclusions	26	Provide a general interpretation of the results in the context of other evidence, and implications for future research.	17
**FUNDING**			
Funding	27	Describe sources of funding for the systematic review and other support (e.g., supply of data); role of funders for the systematic review.	18

For peer review only

PRISMA 2009 Checklist

From: Moher D, Liberati A, Tetzlaff J, Altman DG, The PRISMA Group (2009). Preferred Reporting Items for Systematic Reviews and Meta-Analyses: The PRISMA Statement. PLoS Med 6(6): e1000097. doi:10.1371/journal.pmed1000097

For more information, visit: **www.prisma-statement.org**.

For peer review only

For peer review only - http://bmjopen.bmj.com/site/about/guidelines.xhtml

BMJ Open

Early rehabilitation to prevent post-intensive care syndrome in critically ill patients: a systematic review and meta-analysis

Journal:	*BMJ Open*
Manuscript ID	bmjopen-2017-019998.R2
Article Type:	Research
Date Submitted by the Author:	30-Jan-2018
Complete List of Authors:	Fuke, Ryota; Tohoku Ika Yakka Daigaku Wakabayashi Byoin<br>Hifumi, Toru; Kagawa University Hospital, Emergency Medical Center<br>Kondo, Yutaka; Ryukyu Daigaku,<br>Hatakeyama, Junji; Yokohama Shiritsu Minato Sekijuji Byoin<br>Takei, Tetsuhiro; Yokohama Shiritsu Minato Sekijuji Byoin, Intensive Care Unit<br>Yamakawa, Kazuma; Osaka General Medical Center, Department of Emergency and Critical Care<br>Inoue, Shigeaki; Tokai University Hachioji city Hospital, Department of Emergency and Critical Care Medicine<br>Nishida, Osamu; Fujita Hoken Eisei Daigaku Igakubu Daigakuin Igaku Kenkyuka
**Primary Subject Heading**:	Emergency medicine
Secondary Subject Heading:	Rehabilitation medicine
Keywords:	early rehabilitation, intensive care unit, post-intensive care syndrome, sepsis

SCHOLARONE™
Manuscripts

Early rehabilitation to prevent post-intensive care syndrome in critically ill patients: a systematic review and meta-analysis

Ryota Fuke, MD[1*], Toru Hifumi, MD[2*], Yutaka Kondo, MD, PhD[3], Junji Hatakeyama, MD[4], Tetsuhiro Takei, MD, PhD[4], Kazuma Yamakawa, MD, PhD[5], Shigeaki Inoue, MD, PhD, MSCI[6], Osamu Nishida, MD, PhD[7]

[1] Division of Infectious Diseases and Infection Control, Tohoku Medical and Pharmaceutical University, Sendai City, Miyagi, JAPAN

[2] Emergency Medical Center, Kagawa University Hospital, Kita-gun-Takamatsu, JAPAN

[3] Department of Emergency and Critical Care Medicine, Graduate School of Medicine, University of the Ryukyus, Nakagami-gun, Okinawa, JAPAN

[4] Department of Intensive Care Medicine, Yokohama City Minato Red Cross Hospital, Yokohama City, Kanagawa, JAPAN

[5] Division of Trauma and Surgical Critical Care, Osaka General Medical Center, Osaka City, Osaka, JAPAN

[6] Department of Emergency and Critical Care Medicine, Tokai University Hachioji Hospital, Hachioji, Tokyo, JAPAN

[7] Department of Anaesthesiology and Critical Care Medicine, Fujita Health University School of Medicine, Toyoake City, Aichi, JAPAN

* Ryota Fuke and Toru Hifumi contributed equally to this manuscript.

Address correspondence and reprint requests to: Shigeaki Inoue, Department of Emergency and Critical Care Medicine, Tokai University Hachioji Hospital, Ishikawa-cho 1838, Hachioji City,

For peer review only

For peer review only - http://bmjopen.bmj.com/site/about/guidelines.xhtml

Tokyo 192-0032, Japan. Tel.: +81-42-639-1111; E-mail caf55000@gmail.com

Running title: Early rehabilitation to prevent post-intensive care syndrome

Keywords: early rehabilitation, intensive care unit, post-intensive care syndrome, sepsis

Conflict of interest: The authors declare that they have no conflicts of interest.

Word count: 3032 words

Number of tables: 2

Number of figures: 4

Number of supplementary files for online-only publication: 2

Number of references: 42

For peer review only

ABSTRACT

Introduction: We examined the effectiveness of early rehabilitation for the prevention of post-intensive care syndrome (PICS), characterised by an impaired physical, cognitive, or mental health status, among survivors of critical illness. **Methods**: We performed a systematic literature search of several databases (Medline, Embase, and Cochrane Central Register of Controlled Trials) and a manual search to identify randomised controlled trials (RCTs) comparing the effectiveness of early rehabilitation versus no early rehabilitation or standard care for the prevention of PICS. The primary outcomes were short-term physical-, cognitive-, and mental health-related outcomes assessed during hospitalisation. The secondary outcomes were the standardised, long-term health-related quality of life scores (EuroQol 5 Dimension [EQ5D] and the Medical Outcomes Study 36-Item Short Form Health Survey Physical Function scale [SF-36 PF]). We used the Grading of Recommendations Assessment, Development and Evaluation (GRADE) approach to rate the quality of evidence (QoE). **Results**: Six RCTs selected from 5,105 screened abstracts were included. Early rehabilitation significantly improved short-term physical-related outcomes, as indicated by an increased Medical Research Council scale score (MRC score) [standardised mean difference (SMD): 0.38, 95% confidence interval (CI): 0.10–0.66, p = 0.009] (QoE: low) and a decreased incidence of ICU-acquired weakness (ICU-AW) [odds ratio (OR) 0.42, 95% CI 0.22 to 0.82, p = 0.01] (QoE: low), compared to standard care or no early rehabilitation. However, the two groups did not differ in terms of cognitive-related delirium-free days (SMD: -0.02, 95% CI: -0.23–0.20) (QoE: low) and the mental health-related Hospital Anxiety and Depression Scale score (odds ratio: 0.79, 95% CI: 0.29–2.12) (QoE: low). Early rehabilitation did not improve the long-term outcomes of PICS as characterised by EQ5D and SF-36PF. **Conclusions**: Early rehabilitation improved only short-term physical-related outcomes in critically ill patients. Additional large RCTs are needed.

For peer review only

For peer review only - http://bmjopen.bmj.com/site/about/guidelines.xhtml

Strengths and limitations of this meta-analysis

- ✓ This meta-analysis is the first meta-analysis of comprehensive PICS based on RCTs in which the study intervention populations were limited to early rehabilitation.
- ✓ This meta-analysis was mainly limited by (1) the small number of patients in the included RCTs; (2) a lack of detailed analysis of the adverse effects and PICS domains after hospital discharge; and (3) no confirmation of the exact first day of rehabilitation initiation in three of the eight included RCTs.
- ✓ We used the GRADE approach in the review process.

For peer review only - http://bmjopen.bmj.com/site/about/guidelines.xhtml

INTRODUCTION

Dramatic developments and improvements in the technique, instruments, and education systems used in intensive care units (ICUs) have reduced the mortality of critically ill patients over the past four decades [1]. However, this evolution of life-saving interventions has led to increasing numbers of surviving critically ill patients with an impaired ability to return to physical and mental aspects of normal life [2]. These persistent physical, cognitive and mental impairments experienced by ICU survivors present serious obstacles to discharge from the hospital to a home setting and, once home, to a return to normal daily life [2 3].

Post-intensive care syndrome (PICS) was established as a new syndrome encompassing new or worsening impairments in physical, cognitive, or mental health status that arise after critical illness and persist beyond acute care hospitalisation, with the aim initiating improvements for ICU survivors and their families across the continuum of care [4]. Since the establishment of PICS in 2010, observational studies have evaluated the independent factors associated with this syndrome [5]; however, few intervention studies have targeted the prevention of PICS.

Physiotherapy with early rehabilitation is considered an integral component of the multidisciplinary management of patients in ICUs. In other populations, exercise has been shown to improve strength and function, decrease inflammation [6-8], and affect oxidative stress [9-12], leading to suggestions that early physiotherapy may prevent or reverse some physical impairments in ICU patients. However, no systematic review has investigated the effectiveness of early rehabilitation for the prevention of PICS in ICU patients. The present systematic review aimed to assess the effectiveness of early rehabilitative interventions for the prevention of PICS in ICU patients.

For peer review only

For peer review only - http://bmjopen.bmj.com/site/about/guidelines.xhtml

METHODS

Protocols and registration

This review protocol was registered in PROSPERO, an International Prospective Register of Systematic Reviews at the National Institute for Health Research and Centre for Reviews and Dissemination (CRD) at the University of York (http://www.crd.york.ac.uk/PROSPERO/) (Registration No. CRD42016039759) [13]. This protocol adhered to the Preferred Reporting Items for Systematic Reviews and Meta-Analyses Protocols (PRISMA) statements [14 15], and the systematic review was reported according to PRISMA guidelines [14 16 17]. The protocol for this systematic review was previously published [18].

Search strategy

We searched the Medline (via PubMed from 1996 to 7 June 2016), Embase (until 7 June 2016), and Cochrane Central Register of Controlled Trials (CENTRAL) databases (until 7 June 2016) for full-text clinical trials conducted in humans to retrieve relevant articles for the literature review. We also performed a manual search to retrieve all potentially relevant articles from 8 June to 31 August, 2016. The key search terms used to identify potential studies are listed in Supplementary file 1.

Study selection and inclusion criteria

Three authors (YK, RF, SI) performed the first-line comprehensive literature search and removal of duplicates. Subsequently, two randomly selected authors independently screened the study titles and abstracts for potential relevance. The kappa value for selection bias was 0.396. When disagreements arose between reviewers, the full text of the paper was retrieved, and the disagreements were reconsidered and discussed until a consensus was reached. The full texts of articles included in the final selection were reviewed by two randomly chosen authors. We included trials with the

For peer review only - http://bmjopen.bmj.com/site/about/guidelines.xhtml

following characteristics:

Study types: Randomised controlled trials (RCTs) were included; non-randomised and observational studies were excluded.

Population: Adult patients (aged >18 years) admitted to the ICU were included. We excluded patients with traumatic brain injury and stroke who did not fulfil the PICS criteria, specifically those with acquired physical and psychiatric/cognitive dysfunction after ICU admission.

Intervention: The intervention was early rehabilitation, defined as (1) starting at an earlier time point than usual care or (2) administration within 7 days of ICU admission. 'Rehabilitation' included all physiotherapy, occupational therapy, and palliative care-related support. We excluded RCTs in which rehabilitation was initiated before ICU admission and those comparing early rehabilitation with another intervention.

Control: The control group received standard care or no early rehabilitation.

Outcome: The primary outcomes (i.e., short-term outcomes assessed during hospitalisation) were as follows: (1) physical-related outcomes [incidence of ICU-acquired weakness (AW), Medical Research Council (MRC) [19] scale score], (2) cognitive-related outcomes (delirium-free days); and (3) mental status-related outcomes [Hospital Anxiety and Depression Scale (HADS)] [20]. The secondary outcomes (long-term outcomes assessed post-discharge) included the standardised Health-Related Quality of Life with EuroQol 5 Dimensions (EQ5D) test [21 22] and the Medical Outcomes Study 36-Item Short Form Health Survey Physical Function scale (SF-36 PF) [23] as measures of long-term physical function.

Assessment of the risk of bias

To assess the quality of the included studies, we adopted the Cochrane risk of bias tool [24]. Two randomly selected authors performed the risk of bias assessment; disagreements were resolved by

For peer review only - http://bmjopen.bmj.com/site/about/guidelines.xhtml

discussion, with the inclusion of a third reviewer if necessary. We considered the risk of bias for each element to be 'high' when bias was present and likely to affect outcomes, and 'low' when bias was not present or present but unlikely to affect outcomes [25].

Rating the quality of evidence (QoE) using the GRADE approach

Two authors (RF and TH) independently used the Grading of Recommendations Assessment, Development, and Evaluation (GRADE) tool to rate the quality of evidence (QoE). We used the GRADE approach to rate the QoE of early rehabilitation for outcomes. Although the QoE represents a continuum, we assessed the quality of the body of evidence for each outcome categorised as high, moderate, low, or very low using the GRADE pro Guideline Development Tool.

Data extraction and management

The author(s), title, journal name, year of publication, website URL, and abstract of each included article were identified. Conference abstracts were excluded. Data from each study were extracted independently by two review authors. When faced with insufficient or incomplete data, we contacted the authors of the studies directly. Information related to the study population, sample size, interventions, comparators, potential biases in the conduct of the trial, outcomes (including adverse events), follow-up, and statistical analysis methods were extracted from the original reports into specially designed paper forms and then entered into a spreadsheet.

Summarisation of data and treatment effects

As data from several trials were available, we performed a meta-analysis according to the Cochrane Handbook for Systematic Reviews of Interventions and PRISMA guidelines using Review Manager software (RevMan 5.3, Copenhagen, Denmark: The Nordic Cochrane Centre, the Cochrane

For peer review only - http://bmjopen.bmj.com/site/about/guidelines.xhtml

Collaboration 2014). We summarised the results of this meta-analysis using the generic inverse variance method to facilitate the pooling of estimates of treatment effects. We used odds ratios (ORs) with 95% confidence intervals (CIs) for dichotomous outcomes, and mean differences or standardised mean differences (SMDs) with 95% CIs for continuous outcomes when appropriate. If a quantitative synthesis was not appropriate for a particular outcome, we devised a qualitative summary of that outcome.

Assessment of heterogeneity

For each outcome, we assessed heterogeneity between trials using I^2 statistics to quantify inconsistencies. We considered that significant heterogeneity was present when the reason for heterogeneity could not be explained and the I^2 was ≥50%. The presence of strong clinical heterogeneity was considered when deciding to perform a quantitative data synthesis or a sensitivity analysis with a special focus [26].

Data synthesis

Estimates were pooled using a random effects model. We performed our analysis based on all published or available data.

For peer review only - http://bmjopen.bmj.com/site/about/guidelines.xhtml

RESULTS

Literature search

The PRISMA flow chart of study selection is shown in Figure 1. The combined search strategy identified 5105 citations, of which 46 were judged to be potentially eligible based on the abstract. After excluding 40 of these citations following a full-text review (Supplementary file 2), six RCTs that had enrolled 709 patients were deemed eligible and were included in the review [27-32].

Characteristics of included studies

The study characteristics are summarised in Table 1. Early rehabilitation was initiated within 3 days (median or mean) of ICU admission in five studies [27 29-32]. The first day of rehabilitation was not confirmed in three studies, although early rehabilitation was described in the text, and we contacted the authors to acquire additional information [28]. The rehabilitation strategies administered to each intervention group varied among protocols.

For peer review only

For peer review only - http://bmjopen.bmj.com/site/about/guidelines.xhtml

Table 1. Characteristics of included studies

Source	Population	No. of patients			Age (years)		APACHE II score		Intervention		Timing of first rehabilitation/standard care	Follow-up	Outcome		Ref
		Total	Early Rehab	Control	Early Rehab	Control	Early Rehab	Control	Early Rehab	Control			Short-term (during the hospitalization)	Long-term (post-discharge)	
Brummel 2014	Respiratory failure and/or septic, cardiogenic, or haemorrhagic shock	44	22	22	62 [48–67]	60 [51–69]	21.5 [20.0–28.8]	27.0 [17.5–31.0]	Passive ROM exercises to independent ambulation, guided by the patient's RASS (daily).	Physical therapy (1–2 per week).	Intervention: 1 (1–1), control: 3 (2–6)	3 months	VFD, DFD	EQ5D	29
Hodgson 2016	Critically ill adults mechanically ventilated > 24 h	50	29	21	64 ± 12	53 ± 15	19.8 ± 9.8	15.9 ± 6.9	Functional rehabilitation treatment conducted at the highest level of activity	Not protocolised and all usual unit practice was continued	Intervention: 3 (2–4), control: 3 (2–4)	6 months	MRC, ICU-AW, mechanical ventilation days	EQ5D	33
Jones 2015	Patients ≥ 45 years, had a combined	42	22	20	64 ± 13	60 ± 12	17 ± 10	14 ± 4	In addition to the self-help program, a 6-week	Patient-controlled self-help rehabilita	N/A	3 months	VFD, HAS/HADS		30

For peer review only

For peer review only - http://bmjopen.bmj.com/site/about/guidelines.xhtml

	ICU and pre-ICU stay ≥ 5 days								program of supervised physiotherapy sessions	tion program					
Kayambu 2015	Sepsis patients > 18 years who remained mechanically ventilated > 48 h	50	26	24	62.5 [30–83]	65.5 [37–85]	28 ± 7.6	27 ± 6.8	EMS, passive ROM, active ROM, sitting out of bed, transfers, ambulation for 30 min, one to two times daily	Standard ICU care, which included physical therapy	Within 48 hours of sepsis	6 months	MRC, VFD, HAS/HADS	SF36-PF	31
Morris 2016	Acute respiratory failure patients requiring MV	300	150	150	55 ± 17	58 ± 14	N/A	N/A	Passive ROM, physical therapy, and progressive resistance exercises for 7 days per week	Physical therapy weekday when ordered by the clinical team	Intervention: 1 (0–2), control: 7 (4–10)	6 months	VFD, DFD	SF36-PF	34
Schweickert 2009	Patients who had been on MV < 72 h	104	49	55	57.7 [36.3–69.1]	54.4 [46.5–66.4]	20 [15.8–24]	19 [13.3–23]	Early exercise and physical and occupational therapy on the day of enrolment	Therapy as ordered by the primary care team	Intervention: 1.5 (1.0–2.1), control: 7.4 (6.0–10.9)	N/A	MRC, ICU-AW, VFD, DFD		32

Early Rehab: early rehabilitation, ROM: range of motion, EMS: Electrical muscle stimulation, VFD: ventilator-free days, DFD: delirium -free days , MV: mechanical ventilation

For peer review only - http://bmjopen.bmj.com/site/about/guidelines.xhtml

Risk of bias in the included trials

The evaluation of the risk of bias in each included RCT is shown in the risk of bias summary for PICS-related outcomes without in Figure 2. Regarding blinding, eight trials involved open-label RCTs. The blinding of outcome assessments was not described for 42 RCTs. In all RCTs, attrition bias was observed because of a per-protocol analysis, the inclusion of many patients who dropped out of the study, and death. The performance bias of mortality as an outcome measure was low, however, because blinding did not affect mortality.

Outcomes

The quality assessment and a summary of each outcome measure are shown in Table 2.

For peer review only - http://bmjopen.bmj.com/site/about/guidelines.xhtml

Table 2

Quality assessment and summary of findings for the main comparison

Quality assessment							№ of patients		Effect		Quality	Importance
№ of studies	Study design	Risk of bias	Inconsistency	Indirectness	Imprecision	Other considerations	Early rehabiritation	Control	Relative (95% CI)	Absolute (95% CI)		
Incidence of ICU-AW												
2	randomised trials	serious [a]	not serious	not serious	serious [b]	none	22/78 (28.2%)	37/76 (48.7%)	**OR 0.42** (0.22 to 0.82)	**202 fewer per 1,000** (from 49 fewer to 314 fewer)	⨁⨁◯◯ LOW	CRITICAL
MRC												
3	randomised trials	serious [a]	not serious	not serious	serious [b]	none	97	99	-	SMD **0.38 higher** (0.1 higher to 0.66 higher)	⨁⨁◯◯ LOW	CRITICAL
Delirium-free days												

For peer review only - http://bmjopen.bmj.com/site/about/guidelines.xhtml

Quality assessment							№ of patients		Effect		Quality	Importance
№ of studies	Study design	Risk of bias	Inconsistency	Indirectness	Imprecision	Other considerations	Early rehabiritation	Control	Relative (95% CI)	Absolute (95% CI)		
3	randomised trials	serious [a]	not serious	not serious	serious [b]	none	164	162	-	SMD **0.02 lower** (0.23 lower to 0.2 higher)	⊕⊕◯◯ LOW	CRITICAL
Incidence of HADS/HAS												
2	randomised trials	serious [a]	serious	not serious	serious [b]	none	10/48 (20.8%)	11/44 (25.0%)	**OR 0.79** (0.29 to 2.12)	**42 fewer per 1,000** (from 162 fewer to 164 more)	⊕◯◯◯ VERY LOW	CRITICAL
Health related QOL score (EQ5D)												
2	randomised trials	serious [a]	serious [c]	not serious	serious [b]	none	35	28	-	SMD **0.11 SD higher** (0.86 lower to 1.09 higher)	⊕◯◯◯ VERY LOW	CRITICAL
SF36-PF												

For peer review only - http://bmjopen.bmj.com/site/about/guidelines.xhtml

Quality assessment							№ of patients		Effect		Quality	Importance
№ of studies	Study design	Risk of bias	Inconsistency	Indirectness	Imprecision	Other considerations	Early rehabiritation	Control	Relative (95% CI)	Absolute (95% CI)		
2	randomised trials	serious [a]	not serious	not serious	serious [b]	none	93	98	-	SMD **2.41 higher** (0.75 lower to 5.58 higher)	⊕⊕◯◯ LOW	CRITICAL

CI: Confidence interval; **SMD:** Standardized mean difference; **OR:** Odds ratio

a. Most of the studies are classified as unclear or high risk of bias (performance bias and attrition bias)

b. Confidence intervals for absolute effects are wide and include no effect and important harm and/or small sample size (The number of events is <300)

c. Substantial heterogeneity present

GRADE Working Group grades of evidence High quality: further research is very unlikely to change our confidence in the estimate of effect. Moderate quality: further research is likely to have an important impact on our confidence in the estimate of effect and may change the estimate. Low quality: further research is very likely to have an important impact on our confidence in the estimate of effect and is likely to change the estimate. Very low quality: we are very uncertain about the estimate.

For peer review only - http://bmjopen.bmj.com/site/about/guidelines.xhtml

Primary outcomes (short-term outcomes)

(i) Physical-related outcomes

Incidence of ICU-AW

Two RCTs reported the incidence of ICU-AW (Figure 3). There was a significant reduction in the incidence of ICU-AW in the early rehabilitation group according to the random effects model (two trials, n = 154; OR: 0.42, 95% CI: 0.22–0.82, p = 0.01; I^2 = 0%).

The risk of bias was determined to be serious because all RCTs included a moderate risk of bias. Imprecision was also determined to be serious because of the small number of included patients as per the optimal information size (OIS) criteria. Therefore, the QoE was downgraded from high to low because of these serious risks.

MRC

Three RCTs reported MRC scores (Figure 3). We observed a significant improvement in the early rehabilitation group [MRC (three trials, n = 196; SMD: 0.38, 95% CI: 0.10–0.66, p = 0.009; I^2 = 0%)].

(ii) Cognitive-related outcomes

Delirium-free days

Two RCTs reported the incidence of delirium-free days (Figure 3). There was no significant increase in this parameter in the early rehabilitation group according to a random effects model (two trials, n = 326; SMD: -0.02, 95% CI -0.23–0.20, p = 0.62; I^2 = 0%). The risk of bias was determined to be serious because all RCTs included a moderate risk of bias, and imprecision was also determined to be serious because of the small number of included patients as per OIS criteria. These serious risks

For peer review only - http://bmjopen.bmj.com/site/about/guidelines.xhtml

led to the downgrading of the QoE from high to low.

(iii) Mental health-related outcomes

Incidence of HAS/HADS

Two RCTs reported the incidence of HAS/HADS (Figure 3). These groups did not differ significantly according to a random effects model (two trials, n = 92; OR: 0.79, 95% CI: 0.29–2.12, p = 0.64; I^2 = 0%). The risk of bias was determined to be serious because all RCTs included a moderate risk of bias, and imprecision was also determined to be serious because of the small number of included patients. These serious risks led to the downgrading of the QoE from high to low.

<u>**Secondary outcomes (long-term outcomes)**</u>

EQ5D

Two RCTs reported the EQ5D scores (Figure 4). No significant difference was observed between the two groups according to a random effects model (two trials, n = 63; SMD**:** 0.11, 95% CI: -0.86–1.09, p = 0.82; I^2 = 72%). The risk of bias was deemed serious because all RCTs demonstrated a moderate risk of bias, and inconsistency was deemed serious because of severe heterogeneity (p = 0.06, I^2 = 72%). Imprecision was also considered serious because of the small number of included patients. These serious risks led to the downgrading of the QoE from high to very low.

SF-36 PF

Two RCTs reported SF-36 PF scale scores (Figure 4). However, these scores were evaluated several months later. Moreover, the timing of SF-36 PF evaluation varied greatly among studies (i.e., at hospital discharge and 2, 4, and 6 months after enrolment [32], or at 6 months after hospital discharge

For peer review only - http://bmjopen.bmj.com/site/about/guidelines.xhtml

[29]). We observed a significant improvement in the early rehabilitation group [SF-36 PF (two trials, n = 191; SMD: 2.41, 95% CI: -0.75–5.58, p = 0.14; I^2 = 98%)]. However, the different timings of SF-36 PF scale evaluation among the various studies led to significant heterogeneity. Imprecision was also considered serious because of the small number of included patients as per the OIS criteria. Therefore, these serious risks led to the downgrading of the QoE from high to very low.

For peer review only

For peer review only - http://bmjopen.bmj.com/site/about/guidelines.xhtml

DISCUSSION

Summary of the main results

This meta-analysis revealed that early rehabilitation significantly improved short-term physical function in critically ill patients, as assessed by the MRC scoring system and the incidence of ICU-AW. However, it did not significantly improve the patients' cognitive and mental status-related outcomes. These results suggest that early rehabilitation has a limited effect on the prevention of PICS in critically ill patients, and only reflect improvements in short-term physical function.

Strengths of the review

To our knowledge, the present work is the first meta-analysis of comprehensive PICS based on RCTs in which the study intervention populations were limited to early rehabilitation. Additionally, we used the GRADE approach in the review process, which allowed us to make judgments about the quality of evidence and strength of recommendations in a systematic and transparent manner. Thus, the present meta-analysis allowed us determine whether critical care physicians should initiate early rehabilitation for critically ill patients, and provided new evidence about the effectiveness of physiotherapy interventions aimed at early mobilisation [33].

Agreements and disagreements with other studies or reviews

Four systematic reviews have addressed the effect of rehabilitation [33-35] or early rehabilitation [36] during an ICU stay on functional status. Through a meta-analysis, Kayambu et al. found that physical therapy in the ICU may improve muscle strength, physical function, quality of life, ventilator-free days, ICU stay, and hospital stay; however, the authors included one RCT involving patients who underwent laparotomy (among 10 total RCTs), and another study initiated physical

For peer review only - http://bmjopen.bmj.com/site/about/guidelines.xhtml

therapy 30 days after bed rest [34]. Therefore, the results cannot be directly applied to our clinical practice because of the heterogeneity of the study population. The present meta-analysis showed that early rehabilitation did not improve the patients' quality of life. The inconsistency of these results might be attributable to differences in the methods used to assess quality of life. Kayambu et al. used the Medical Outcomes Study 36-Item Short Form survey score, whereas the present review included used the EQ5D.

Castro-Avila et al. conducted a systematic review and meta-analysis of the effect of early rehabilitation during an ICU stay on functional status [34], and included the study reported by Denehy et al [37]. However, we did not include this study in our systematic review, as it implemented early active rehabilitation in the control group. This implementation was the main reason underlying the authors' conclusion that early rehabilitation was not associated with improvements in the walking distance and incidence of ICU-AW. Furthermore, Tipping et al. reported a systematic review regarding the effects of active mobilisation and rehabilitation in the ICU on mortality and function [35], and included a study by Moss et al [38]. However, that study did not initiate rehabilitation during the early phase and therefore was also not included in the current systematic review.

Clinical implementation

The initiation of early rehabilitation improved short-term outcomes, such as the physical function score and the incidence of ICU-AW in the present systematic review; however, it did not improve. The tendency to favour early rehabilitation during mechanical ventilation suggests that early rehabilitation could potentially improve short-term survival and maintain and increase muscle strength.

However, the initiation of rehabilitation during acute phase may inflict tremendous physical stress and exhaustion upon patients in the ICU, thus increasing ICU mortality [39]. Although several

For peer review only - http://bmjopen.bmj.com/site/about/guidelines.xhtml

For peer review only

studies included in the present meta-analysis described the safety of early physiotherapy in the ICU [27 29-32 40], only two outcomes related to the safety of early rehabilitation, the blood lactate level [29] and early physiotherapy termination rate [31], were compared between two groups. Therefore, the study conclusions should be approached with caution.

Limitations of the review

The current **meta-analysis** had several limitations. First, the analysis of each outcome included only a small number of patients. Although Schweickert et al. reported data concerning delirium, we could not extract cognitive-related outcomes because no data about delirium-free days were not addressed directly [30]. Second, the studies included in the current systematic review did not measure the domains of PICS beyond hospital discharge. Third, two of the included studies were pilot/feasibility trials and not powered to identify differences in physical or cognitive outcomes [27 31]. However, it was reasonable to initiate RCTs in the context of feasibility studies because no large multicentre trials of the effects of early mobilisation in the ICU had been published before the study conducted by Brummel et al., and little evidence to support the feasibility of individual patient randomisation across multiple sites using a complex intervention (i.e. early mobilisation) had been reported before the study conducted by Hodgson et al. Moreover, these two RCTs demonstrated the same level of risk as other RCTs, according to risk of bias summary conducted in this meta-analysis (Figure 2). Fourth, we could not confirm the exact first day of rehabilitation initiation in the three studies, although 'early rehabilitation' was described in the text and we contacted the authors to acquire additional information [28 40]. Fifth, we could not find any data regarding direct cognitive assessment in the selected papers. Although cognitive impairment at hospital discharge may not predict long-term cognitive impairment [41], Pandharipande et al. reported that a longer duration of delirium upon admission was associated with worse global cognition and executive function scores in the

For peer review only - http://bmjopen.bmj.com/site/about/guidelines.xhtml

surgical ICU at 3 and 12 months [42]. Therefore, we believe that the assessment of delirium in the ICU and hospital is a very important predictor of long-term cognitive impairment post-discharge. Sixth, as this meta-analysis included studies that had implemented various 'early rehabilitation' protocols, further analyses should include large trials with strict and comparable rehabilitation algorithms adjusted for the initiation time, type, and intensity. Despite these several limitations, our meta-analysis clarifies the effectiveness of early rehabilitation for the prevention of PICS in survivors of critical illness.

CONCLUSION

Early rehabilitation has a limited effect on the prevention of PICS, although it led to significant improvements in short-term physical-related outcomes, including MRC scores and the incidence of ICU-AW. However, early rehabilitation had no significant effect on cognitive function and mental health-related outcomes or mortality in critically ill patients. Additional large-scale, rigorous RCTs are needed to confirm our results.

For peer review only

For peer review only - http://bmjopen.bmj.com/site/about/guidelines.xhtml

Contributors

YK, RF, TH, JH, TT, and SI conceived the idea for this systematic review. YK, RF, TH, JH, TT, and SI developed the methodology for the systematic review, and KY and ON supervised the methodological process. The manuscript was drafted by YK and SI. RF, TH, JH, TT, and KY and revised by ON. All authors critically reviewed and approved the final manuscript.

Funding

No funding support was received for this systematic review meta-analysis.

Competing interests

None declared.

Provenance and peer review

This work was not commissioned and was externally peer reviewed.

Data sharing statement Extra data can be accessed via the Dryad data repository at http://datadryad.org/ with the doi:10.5061/dryad.12q6c78

For peer review only

FIGURE LEGENDS

Figure 1

Flow chart of the process of study identification and inclusion

Figure 2

Risk of bias summary

Figure 3

The effect of early rehabilitation on short-term outcomes in post-intensive care syndrome (PICS) in intensive care unit (ICU) patients.1) Physical-related outcomes **A.** Incidence of ICU-acquired weakness (AW). **B.** Medical Research Council sum score. 2) Cognitive-related outcomes, characterised by delirium-free days. 3) Mental status-related outcomes, characterised by the Hospital Anxiety and Depression Scale (HAS/HADS) score. CI, confidence interval

Figure 4

The effect of early rehabilitation on long-term outcomes in post-intensive care syndrome (PICS) in intensive care unit (ICU) patients. The effect of early rehabilitation on health-related quality of life (QOL) scores and in ICU patients. 1) Health-related quality of life (QOL) scores calculated from the EuroQol 5 Dimensions (EQ5D). 2) Medical Outcomes Study 36-Item Short Form Health Survey Physical Function scale (SF-36 PF).

For peer review only

For peer review only - http://bmjopen.bmj.com/site/about/guidelines.xhtml

REFERENCES

1. Bemis-Dougherty AR, Smith JM. What follows survival of critical illness? Physical therapists' management of patients with post-intensive care syndrome. *Phys Ther* 2013;93(2):179-85. doi: 10.2522/ptj.20110429

2. Iwashyna TJ, Ely EW, Smith DM, et al. Long-term cognitive impairment and functional disability among survivors of severe sepsis. *JAMA : the journal of the American Medical Association* 2010;304(16):1787-94. doi: 10.1001/jama.2010.1553

3. Desai SV, Law TJ, Needham DM. Long-term complications of critical care. *Crit Care Med* 2011;39(2):371-9. doi: 10.1097/CCM.0b013e3181fd66e5

4. Needham DM, Davidson J, Cohen H, et al. Improving long-term outcomes after discharge from intensive care unit: report from a stakeholders' conference. *Crit Care Med* 2012;40(2):502-9. doi: 10.1097/CCM.0b013e318232da75

5. Elliott D, Davidson JE, Harvey MA, et al. Exploring the scope of post-intensive care syndrome therapy and care: engagement of non-critical care providers and survivors in a second stakeholders meeting. *Crit Care Med* 2014;42(12):2518-26. doi: 10.1097/CCM.0000000000000525

6. Gielen S, Adams V, Möbius-Winkler S, et al. Anti-inflammatory effects of exercise training in the skeletal muscle of patients with chronic heart failure. *J Am Coll Cardiol* 2003;42(5):861-68. doi: 10.1016/S0735-1097(03)00848-9

7. Nuhr MJ, Pette D, Berger R, et al. Beneficial effects of chronic low-frequency stimulation of thigh muscles in patients with advanced chronic heart failure. *Eur Heart J* 2004;25(2):136-43. doi: 10.1016/j.ehj.2003.09.027

8. Vivodtzev I, Pépin JL, Vottero G, et al. Improvement in quadriceps strenght and dyspnea in daily tasks after 1 month of electrical stimulation in severely deconditioned and malnourished COPD. *Chest* 2006;129(6):1540-48. doi: 10.1378/chest.129.6.1540

9. Laaksonen DE, Atalay M, Niskanen L, et al. Increased resting and exercise-induced oxidative stress in young IDDM men. *Diabetes care* 1996;19(6):569-74.

10. Mercken EM, Hageman GJ, Schols AMWJ, et al. Rehabilitation decreases exercise-induced oxidative stress in chronic obstructive pulmonary disease. *American Journal of Respiratory and Critical Care Medicine* 2005;172(8):994-1001. doi: 10.1164/rccm.200411-1580OC

11. Nishiyama Y, Ikeda H, Haramaki N, et al. Oxidative stress is related to exercise intolerance in patients with heart failure. *Am Heart J* 1998;135(1):115-20. doi: 10.1016/S0002-8703(98)70351-5

12. Vincent HK, Morgan JW, Vincent KR. Obesity Exacerbates Oxidative Stress Levels after Acute Exercise. *Med Sci Sports Exerc* 2004;36(5):772-79.

For peer review only

For peer review only - http://bmjopen.bmj.com/site/about/guidelines.xhtml

13. Moher D, Booth A, Stewart L. How to reduce unnecessary duplication: use PROSPERO. *BJOG : an international journal of obstetrics and gynaecology* 2014;121(7):784-6. doi: 10.1111/1471-0528.12657

14. Moher D, Shamseer L, Clarke M, et al. Preferred reporting items for systematic review and meta-analysis protocols (PRISMA-P) 2015 statement. *Systematic reviews* 2015;4:1. doi: 10.1186/2046-4053-4-1

15. Shamseer L, Moher D, Clarke M, et al. Preferred reporting items for systematic review and meta-analysis protocols (PRISMA-P) 2015: elaboration and explanation. *BMJ* 2015;349:g7647. doi: 10.1136/bmj.g7647

16. Liberati A, Altman DG, Tetzlaff J, et al. The PRISMA statement for reporting systematic reviews and meta-analyses of studies that evaluate health care interventions: explanation and elaboration. *J Clin Epidemiol* 2009;62(10):e1-34. doi: 10.1016/j.jclinepi.2009.06.006

17. Moher D, Liberati A, Tetzlaff J, et al. Preferred reporting items for systematic reviews and meta-analyses: the PRISMA statement. *PLoS medicine* 2009;6(7):e1000097. doi: 10.1371/journal.pmed.1000097

18. Kondo Y, Fuke R, Hifumi T, et al. Early rehabilitation for the prevention of postintensive care syndrome in critically ill patients: a study protocol for a systematic review and meta-analysis. *BMJ open* 2017;7(3):e013828. doi: 10.1136/bmjopen-2016-013828

19. Paternostro-Sluga T, Grim-Stieger M, Posch M, et al. Reliability and validity of the Medical Research Council (MRC) scale and a modified scale for testing muscle strength in patients with radial palsy. *Journal of rehabilitation medicine* 2008;40(8):665-71. doi: 10.2340/16501977-0235

20. Yang ZZ, Grote DM, Xiu B, et al. TGF-beta upregulates CD70 expression and induces exhaustion of effector memory T cells in B-cell non-Hodgkin's lymphoma. *Leukemia : official journal of the Leukemia Society of America, Leukemia Research Fund, UK* 2014;28(9):1872-84. doi: 10.1038/leu.2014.84

21. Mozzi A, Meregaglia M, Lazzaro C, et al. A comparison of EuroQol 5-Dimension health-related utilities using Italian, UK, and US preference weights in a patient sample. *Clinicoecon Outcomes Res* 2016;8:267-74. doi: 10.2147/CEOR.S98226

22. Obradovic M, Lal A, Liedgens H. Validity and responsiveness of EuroQol-5 dimension (EQ-5D) versus Short Form-6 dimension (SF-6D) questionnaire in chronic pain. *Health Qual Life Outcomes* 2013;11:110. doi: 10.1186/1477-7525-11-110

23. Yang X, Fan D, Xia Q, et al. The health-related quality of life of ankylosing spondylitis patients assessed by SF-36: a systematic review and meta-analysis. *Qual Life Res* 2016;25(11):2711-23. doi: 10.1007/s11136-016-1345-z

For peer review only

For peer review only - http://bmjopen.bmj.com/site/about/guidelines.xhtml

24. JPT H. Chapter 8: Assessing risk of bias in included studies. 2011

25. Duan EH, Oczkowski SJ, Belley-Cote E, et al. beta-Blockers in sepsis: protocol for a systematic review and meta-analysis of randomised control trials. *BMJ open* 2016;6(6):e012466. doi: 10.1136/bmjopen-2016-012466

26. Klotz R, Klaiber U, Grummich K, et al. Percutaneous versus surgical strategy for tracheostomy: protocol for a systematic review and meta-analysis of perioperative and postoperative complications. *Systematic reviews* 2015;4:105. doi: 10.1186/s13643-015-0092-5

27. Brummel NE, Girard TD, Ely EW, et al. Feasibility and safety of early combined cognitive and physical therapy for critically ill medical and surgical patients: the Activity and Cognitive Therapy in ICU (ACT-ICU) trial. *Intensive Care Med* 2014;40(3):370-9. doi: 10.1007/s00134-013-3136-0

28. Jones C, Eddleston J, McCairn A, et al. Improving rehabilitation after critical illness through outpatient physiotherapy classes and essential amino acid supplement: A randomized controlled trial. *Journal of critical care* 2015 doi: 10.1016/j.jcrc.2015.05.002

29. Kayambu G, Boots R, Paratz J. Early physical rehabilitation in intensive care patients with sepsis syndromes: a pilot randomised controlled trial. *Intensive Care Med* 2015;41(5):865-74. doi: 10.1007/s00134-015-3763-8

30. Schweickert WD, Pohlman MC, Pohlman AS, et al. Early physical and occupational therapy in mechanically ventilated, critically ill patients: a randomised controlled trial. *Lancet* 2009;373(9678):1874-82. doi: 10.1016/S0140-6736(09)60658-9

31. Hodgson CL, Bailey M, Bellomo R, et al. A Binational Multicenter Pilot Feasibility Randomized Controlled Trial of Early Goal-Directed Mobilization in the ICU. *Crit Care Med* 2016;44(6):1145-52. doi: 10.1097/CCM.0000000000001643

32. Morris PE, Berry MJ, Files DC, et al. Standardized Rehabilitation and Hospital Length of Stay Among Patients With Acute Respiratory Failure: A Randomized Clinical Trial. *JAMA : the journal of the American Medical Association* 2016;315(24):2694-702. doi: 10.1001/jama.2016.7201

33. Stiller K. Physiotherapy in intensive care: an updated systematic review. *Chest* 2013;144(3):825-47. doi: 10.1378/chest.12-2930

34. Kayambu G, Boots R, Paratz J. Physical therapy for the critically ill in the ICU: a systematic review and meta-analysis. *Crit Care Med* 2013;41(6):1543-54. doi: 10.1097/CCM.0b013e31827ca637

35. Tipping CJ, Harrold M, Holland A, et al. The effects of active mobilisation and rehabilitation in ICU on mortality and function: a systematic review. *Intensive Care Med* 2016 doi: 10.1007/s00134-016-4612-0

36. Castro-Avila AC, Seron P, Fan E, et al. Effect of Early Rehabilitation during Intensive Care Unit Stay

For peer review only

For peer review only - http://bmjopen.bmj.com/site/about/guidelines.xhtml

on Functional Status: Systematic Review and Meta-Analysis. *PloS one* 2015;10(7):e0130722. doi: 10.1371/journal.pone.0130722

37. Denehy L, Skinner EH, Edbrooke L, et al. Exercise rehabilitation for patients with critical illness: a randomized controlled trial with 12 months of follow-up. *Crit Care* 2013;17(4):R156. doi: 10.1186/cc12835 [published Online First: 2013/07/26]

38. Moss M, Nordon-Craft A, Malone D, et al. A Randomized Trial of an Intensive Physical Therapy Program for Patients with Acute Respiratory Failure. *Am J Respir Crit Care Med* 2016;193(10):1101-10. doi: 10.1164/rccm.201505-1039OC

39. Greening NJ, Williams JE, Hussain SF, et al. An early rehabilitation intervention to enhance recovery during hospital admission for an exacerbation of chronic respiratory disease: randomised controlled trial. *BMJ* 2014;349:g4315. doi: 10.1136/bmj.g4315

40. Cameron JI, Chu LM, Matte A, et al. One-Year Outcomes in Caregivers of Critically Ill Patients. *The New England journal of medicine* 2016;374(19):1831-41. doi: 10.1056/NEJMoa1511160

41. Woon FL, Dunn CB, Hopkins RO. Predicting cognitive sequelae in survivors of critical illness with cognitive screening tests. *Am J Respir Crit Care Med* 2012;186(4):333-40. doi: 10.1164/rccm.201112-2261OC

42. Pandharipande PP, Girard TD, Jackson JC, et al. Long-term cognitive impairment after critical illness. *The New England journal of medicine* 2013;369(14):1306-16. doi: 10.1056/NEJMoa1301372

For peer review only

For peer review only - http://bmjopen.bmj.com/site/about/guidelines.xhtml

Figure 1

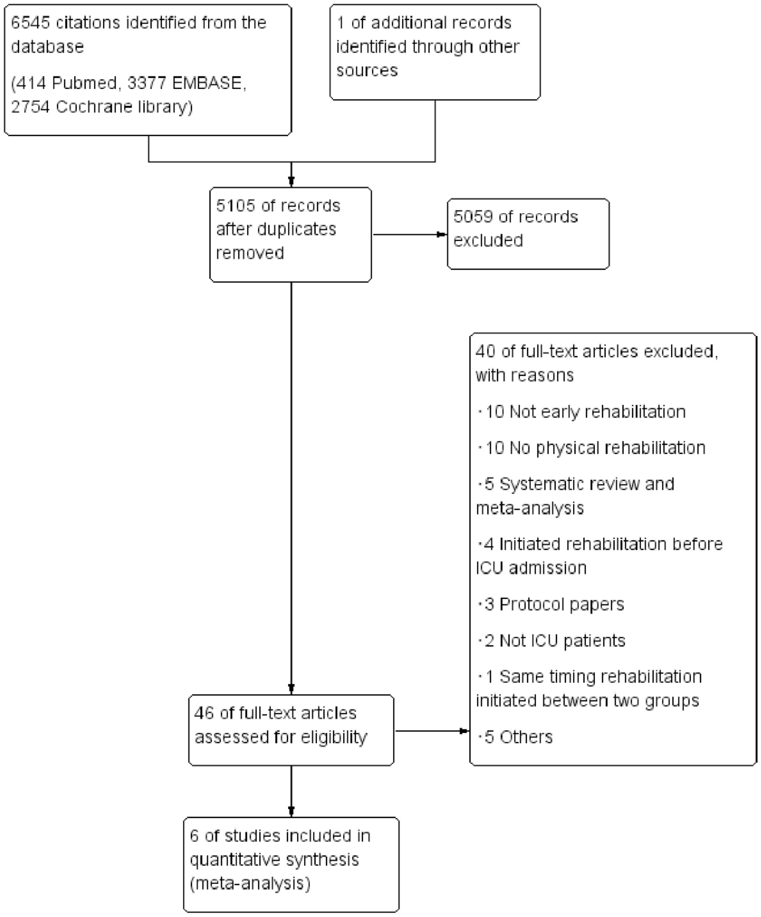

Figure 1

254x338mm (300 x 300 DPI)

Figure 2

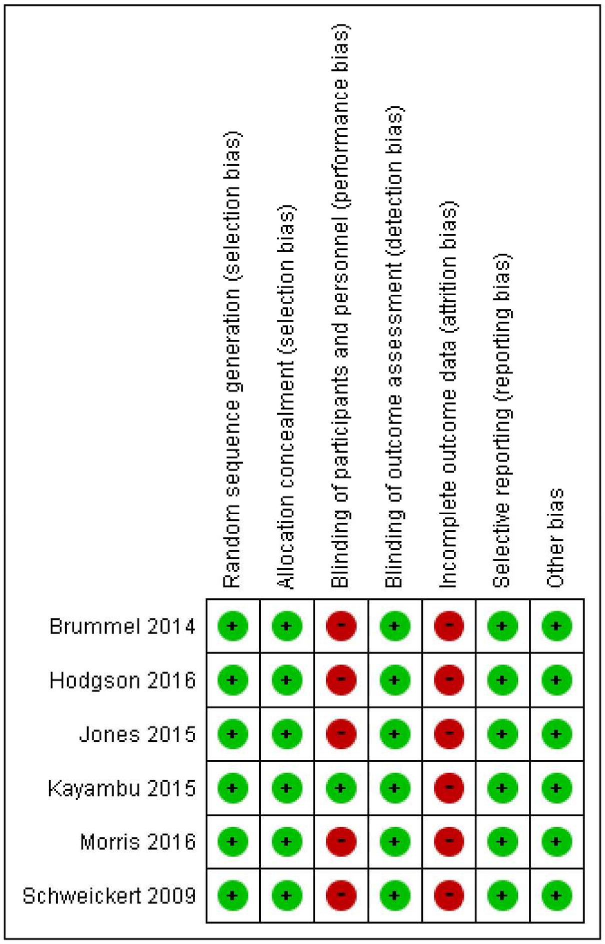

Figure 2

254x338mm (300 x 300 DPI)

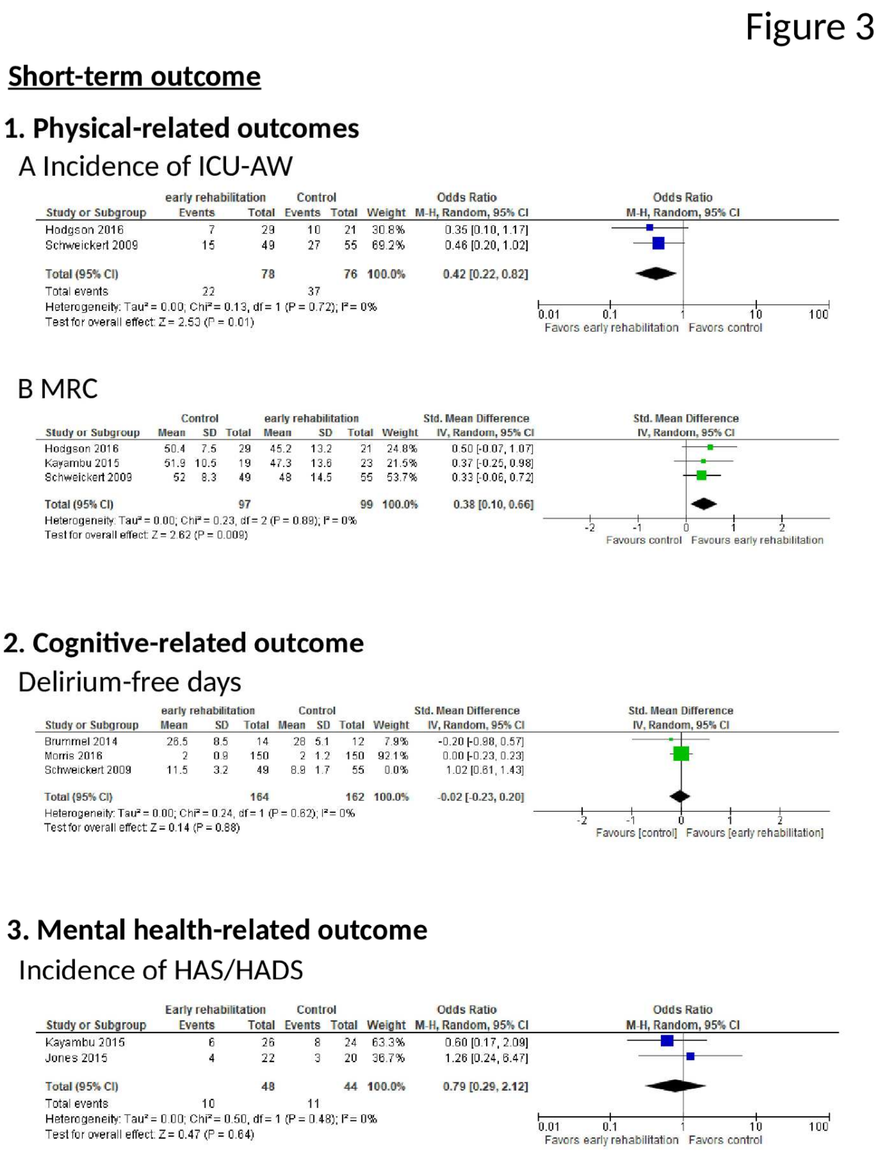

Figure 3

254x338mm (300 x 300 DPI)

Figure 4

Long-term outcome

1 Health-related QOL scores

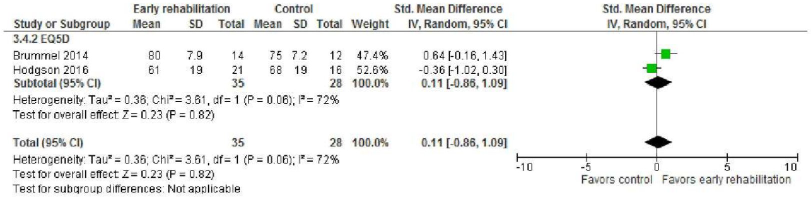

2 SF-36PF

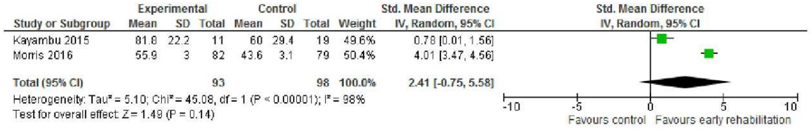

Figure 4

254x338mm (300 x 300 DPI)

Supplementary file 1.

The full search strategies for Medline (via PubMed), EMBASE and the Cochrane Central Register of clinical trials.

#1 ("critical ill" or "critical illness" or "critical care" or "intensive care" or "mechanical ventilation" or "mechanical ventilated" or "postoperative care")

#2 (rehabilitation or "physical therapy" or physiotherapy or exercise or mobilization or "mobility intervention" or "muscle training")

#3 ("Activities of Daily Living" or "Quality of Life" or "post-intensive care syndrome" or "motor function" or "Physical Functioning" or "functional status" or "physical function" or "ventilator days" or "quality of life" or (walking or walk) or muscle or polyneuromyopathy' or "length of stay" or "length of ICU stay" or "length of hospital stay" or "intubation period" or "duration of mechanical ventilation" or re-admission or "functional outcome" or "ICU-acquired weakness" or "ICU-acquired paresis" or ICUAW or "ICU-AW" or "intensive care unit acquired weakness" or "critical illness polyneuropathy" or "critical illness myopathy" or "critical illness neuromyopathy" or "acute quadriplegic myopathy" or "thick filament myopathy" or "acute necrotizing myopathy of intensive care" or "acute corticosteroid myopathy" or "critical illness neuromuscula syndromes" or "Tower test" or "Timed Up and Go Test" or "dysexecutive questionnaire" or FAQ or "EQ-5D VAS" or 6MWD or "6-min walking distance" or "Quadriceps force, and self-perceived functional status" or "SF-36 PF" or MRC or "Medical Research Council" or "AQoL utility" or "EQ-5D" or PFIT or "physical functional ICU test" or "Hospital Anxiety and Depression Scale" or "Hand-grip strength")

#4 #1 AND #2 AND #3

Article search was performed at 7 July 2016 and a total of 5104 articles are scanned.

For peer review only

Supplementary file 2

List of excluded studies

40 excluded studies with reasons

10 No early rehabilitation [1-10]

10 No physical rehabilitation [11-20]

5 Systematic review and meta-analysis [21-25]

4 Initiated rehabilitation before ICU admission [26-29]

3 Protocol paper [30-32]

2 Not ICU patients [33 34]

1 Same timing rehabilitation initiated between two groups [35]

5 Others [36-40]

For peer review only

References

1. Stein R, Maia CP, Silveira AD, et al. Inspiratory muscle strength as a determinant of functional capacity early after coronary artery bypass graft surgery. *Archives of physical medicine and rehabilitation* 2009;90(10):1685-91. doi: 10.1016/j.apmr.2009.05.010
2. Patman S, Sanderson D, Blackmore M. Physiotherapy following cardiac surgery: is it necessary during the intubation period? *The Australian journal of physiotherapy* 2001;47(1):7-16.
3. Porta R, Vitacca M, Gile LS, et al. Supported arm training in patients recently weaned from mechanical ventilation. *Chest* 2005;128(4):2511-20. doi: 10.1378/chest.128.4.2511
4. Jackson JC, Ely EW, Morey MC, et al. Cognitive and physical rehabilitation of intensive care unit survivors: results of the RETURN randomized controlled pilot investigation. *Crit Care Med* 2012;40(4):1088-97. doi: 10.1097/CCM.0b013e3182373115
5. Patman S, Jenkins S, Stiller K. Physiotherapy does not prevent, or hasten recovery from, ventilator-associated pneumonia in patients with acquired brain injury. *Intensive Care Med* 2009;35(2):258-65. doi: 10.1007/s00134-008-1278-2
6. Chen S, Su CL, Wu YT, et al. Physical training is beneficial to functional status and survival in patients

with prolonged mechanical ventilation. *Journal of the Formosan Medical Association = Taiwan yi zhi* 2011;110(9):572-9. doi: 10.1016/j.jfma.2011.07.008

7. Walsh TS, Salisbury LG, Merriweather JL, et al. Increased Hospital-Based Physical Rehabilitation and Information Provision After Intensive Care Unit Discharge: The RECOVER Randomized Clinical Trial. *JAMA internal medicine* 2015;175(6):901-10. doi: 10.1001/jamainternmed.2015.0822
8. Chiang LL, Wang LY, Wu CP, et al. Effects of physical training on functional status in patients with prolonged mechanical ventilation. *Phys Ther* 2006;86(9):1271-81. doi: 10.2522/ptj.20050036
9. Chen YH, Lin HL, Hsiao HF, et al. Effects of exercise training on pulmonary mechanics and functional status in patients with prolonged mechanical ventilation. *Respir Care* 2012;57(5):727-34. doi: 10.4187/respcare.01341
10. Burtin C, Clerckx B, Robbeets C, et al. Early exercise in critically ill patients enhances short-term functional recovery. *Crit Care Med* 2009;37(9):2499-505. doi: 10.1097/CCM.0b013e3181a38937
11. Jackson JC, Girard TD, Gordon SM, et al. Long-term cognitive and psychological outcomes in the awakening and breathing controlled trial. *Am J Respir Crit Care Med* 2010;182(2):183-91. doi: 10.1164/rccm.200903-0442OC
12. Nava S. Rehabilitation of patients admitted to a respiratory intensive care unit. *Archives of physical medicine and rehabilitation* 1998;79(7):849-54.
13. Mackay MR, Ellis E, Johnston C. Randomised clinical trial of physiotherapy after open abdominal surgery in high risk patients. *The Australian journal of physiotherapy* 2005;51(3):151-9.
14. Stiller K, Montarello J, Wallace M, et al. Efficacy of breathing and coughing exercises in the prevention of pulmonary complications after coronary artery surgery. *Chest* 1994;105(3):741-7.
15. Caruso P, Denari SD, Ruiz SA, et al. Inspiratory muscle training is ineffective in mechanically ventilated critically ill patients. *Clinics (Sao Paulo)* 2005;60(6):479-84. doi: /S1807-59322005000600009
16. Albert NM, Gillinov AM, Lytle BW, et al. A randomized trial of massage therapy after heart surgery. *Heart Lung* 2009;38(6):480-90. doi: 10.1016/j.hrtlng.2009.03.001
17. Pattanshetty RB, Gaude GS. Effect of multimodality chest physiotherapy on the rate of recovery and prevention of complications in patients with mechanical ventilation: a prospective study in medical and surgical intensive care units. *Indian J Med Sci* 2011;65(5):175-85. doi: 10.4103/0019-5359.106608
18. Templeton M, Palazzo MG. Chest physiotherapy prolongs duration of ventilation in the critically ill ventilated for more than 48 hours. *Intensive Care Med* 2007;33(11):1938-45. doi: 10.1007/s00134-007-0762-4
19. Cader SA, Vale RG, Castro JC, et al. Inspiratory muscle training improves maximal inspiratory pressure and may assist weaning in older intubated patients: a randomised trial. *Journal of physiotherapy* 2010;56(3):171-7.

For peer review only

20. Pattanshetty RB, Gaude GS. Effect of multimodality chest physiotherapy in prevention of ventilator-associated pneumonia: A randomized clinical trial. *Indian journal of critical care medicine : peer-reviewed, official publication of Indian Society of Critical Care Medicine* 2010;14(2):70-6. doi: 10.4103/0972-5229.68218

21. Patel BK, Pohlman AS, Hall JB, et al. Impact of early mobilization on glycemic control and ICU-acquired weakness in critically ill patients who are mechanically ventilated. *Chest* 2014;146(3):583-9. doi: 10.1378/chest.13-2046

22. Connolly B, Salisbury L, O'Neill B, et al. Exercise rehabilitation following intensive care unit discharge for recovery from critical illness. *Cochrane Database Syst Rev* 2015(6):CD008632. doi: 10.1002/14651858.CD008632.pub2

23. Mehrholz J, Pohl M, Kugler J, et al. Physical rehabilitation for critical illness myopathy and neuropathy. *Cochrane Database Syst Rev* 2015(3):CD010942. doi: 10.1002/14651858.CD010942.pub2

24. Elkins M, Dentice R. Inspiratory muscle training facilitates weaning from mechanical ventilation among patients in the intensive care unit: a systematic review. *Journal of physiotherapy* 2015;61(3):125-34. doi: 10.1016/j.jphys.2015.05.016

25. Castro-Avila AC, Seron P, Fan E, et al. Effect of Early Rehabilitation during Intensive Care Unit Stay on Functional Status: Systematic Review and Meta-Analysis. *PloS one* 2015;10(7):e0130722. doi: 10.1371/journal.pone.0130722

26. Herdy AH, Marcchi PL, Vila A, et al. Pre- and postoperative cardiopulmonary rehabilitation in hospitalized patients undergoing coronary artery bypass surgery: a randomized controlled trial. *Am J Phys Med Rehabil* 2008;87(9):714-9. doi: 10.1097/PHM.0b013e3181839152

27. Arthur HM, Daniels C, McKelvie R, et al. Effect of a preoperative intervention on preoperative and postoperative outcomes in low-risk patients awaiting elective coronary artery bypass graft surgery. A randomized, controlled trial. *Annals of internal medicine* 2000;133(4):253-62.

28. Fagevik Olsen M, Hahn I, Nordgren S, et al. Randomized controlled trial of prophylactic chest physiotherapy in major abdominal surgery. *Br J Surg* 1997;84(11):1535-8.

29. Weiner P, Zeidan F, Zamir D, et al. Prophylactic inspiratory muscle training in patients undergoing coronary artery bypass graft. *World journal of surgery* 1998;22(5):427-31.

30. Kayambu G, Boots RJ, Paratz JD. Early rehabilitation in sepsis: a prospective randomised controlled trial investigating functional and physiological outcomes The i-PERFORM Trial (Protocol Article). *BMC anesthesiology* 2011;11:21. doi: 10.1186/1471-2253-11-21

31. Thomas K, Wright SE, Watson G, et al. Extra Physiotherapy in Critical Care (EPICC) Trial Protocol: a randomised controlled trial of intensive versus standard physical rehabilitation therapy in the critically ill. *BMJ open* 2015;5(5):e008035. doi: 10.1136/bmjopen-2015-008035

32. Brummel NE, Jackson JC, Girard TD, et al. A combined early cognitive and physical rehabilitation

For peer review only

program for people who are critically ill: the activity and cognitive therapy in the intensive care unit (ACT-ICU) trial. *Phys Ther* 2012;92(12):1580-92. doi: 10.2522/ptj.20110414

33. Reeve JC, Nicol K, Stiller K, et al. Does physiotherapy reduce the incidence of postoperative pulmonary complications following pulmonary resection via open thoracotomy? A preliminary randomised single-blind clinical trial. *Eur J Cardiothorac Surg* 2010;37(5):1158-66. doi: 10.1016/j.ejcts.2009.12.011
34. Lee SM, Kang SB, Jang JH, et al. Early rehabilitation versus conventional care after laparoscopic rectal surgery: a prospective, randomized, controlled trial. *Surgical endoscopy* 2013;27(10):3902-9. doi: 10.1007/s00464-013-3006-4
35. Hirschhorn AD, Richards DA, Mungovan SF, et al. Does the mode of exercise influence recovery of functional capacity in the early postoperative period after coronary artery bypass graft surgery? A randomized controlled trial. *Interactive cardiovascular and thoracic surgery* 2012;15(6):995-1003. doi: 10.1093/icvts/ivs403
36. Yosef-Brauner O, Adi N, Ben Shahar T, et al. Effect of physical therapy on muscle strength, respiratory muscles and functional parameters in patients with intensive care unit-acquired weakness. *The clinical respiratory journal* 2015;9(1):1-6. doi: 10.1111/crj.12091
37. Denehy L, Skinner EH, Edbrooke L, et al. Exercise rehabilitation for patients with critical illness: a randomized controlled trial with 12 months of follow-up. *Crit Care* 2013;17(4):R156. doi: 10.1186/cc12835
38. Melchers P, Maluck A, Suhr L, et al. An early onset rehabilitation program for children and adolescents after traumatic brain injury (TBI): Methods and first results. *Restorative neurology and neuroscience* 1999;14(2):153-60.
39. Dantas CM, Silva PF, Siqueira FH, et al. Influence of early mobilization on respiratory and peripheral muscle strength in critically ill patients. *Revista brasileira de terapia intensiva* 2012;24(2):173-8.
40. Dong ZH, Yu BX, Sun YB, et al. Effects of early rehabilitation therapy on patients with mechanical ventilation. *World journal of emergency medicine* 2014;5(1):48-52. doi: 10.5847/wjem.j.1920-8642.2014.01.008

For peer review only

PRISMA 2009 Checklist

Section/topic	#	Checklist item	Reported on page #
**TITLE**			
Title	1	Identify the report as a systematic review, meta-analysis, or both.	1
**ABSTRACT**			
Structured summary	2	Provide a structured summary including, as applicable: background; objectives; data sources; study eligibility criteria, participants, and interventions; study appraisal and synthesis methods; results; limitations; conclusions and implications of key findings; systematic review registration number.	3
**INTRODUCTION**			
Rationale	3	Describe the rationale for the review in the context of what is already known.	5
Objectives	4	Provide an explicit statement of questions being addressed with reference to participants, interventions, comparisons, outcomes, and study design (PICOS).	5
**METHODS**			
Protocol and registration	5	Indicate if a review protocol exists, if and where it can be accessed (e.g., Web address), and, if available, provide registration information including registration number.	6
Eligibility criteria	6	Specify study characteristics (e.g., PICOS, length of follow-up) and report characteristics (e.g., years considered, language, publication status) used as criteria for eligibility, giving rationale.	6, 7
Information sources	7	Describe all information sources (e.g., databases with dates of coverage, contact with study authors to identify additional studies) in the search and date last searched.	6, 8
Search	8	Present full electronic search strategy for at least one database, including any limits used, such that it could be repeated.	Suppl 1
Study selection	9	State the process for selecting studies (i.e., screening, eligibility, included in systematic review, and, if applicable, included in the meta-analysis).	7, 8, 9
Data collection process	10	Describe method of data extraction from reports (e.g., piloted forms, independently, in duplicate) and any processes for obtaining and confirming data from investigators.	8
Data items	11	List and define all variables for which data were sought (e.g., PICOS, funding sources) and any assumptions and simplifications made.	-
Risk of bias in individual studies	12	Describe methods used for assessing risk of bias of individual studies (including specification of whether this was done at the study or outcome level), and how this information is to be used in any data synthesis.	7, 8
Summary measures	13	State the principal summary measures (e.g., risk ratio, difference in means).	8, 9
Synthesis of results	14	Describe the methods of handling data and combining results of studies, if done, including measures of consistency (e.g., I^2) for each meta-analysis.	8, 9

For peer review only - http://bmjopen.bmj.com/site/about/guidelines.xhtml

PRISMA 2009 Checklist

Section/topic	#	Checklist item	Reported on page #
Risk of bias across studies	15	Specify any assessment of risk of bias that may affect the cumulative evidence (e.g., publication bias, selective reporting within studies).	9
Additional analyses	16	Describe methods of additional analyses (e.g., sensitivity or subgroup analyses, meta-regression), if done, indicating which were pre-specified.	-
**RESULTS**			
Study selection	17	Give numbers of studies screened, assessed for eligibility, and included in the review, with reasons for exclusions at each stage, ideally with a flow diagram.	10, Figure 1
Study characteristics	18	For each study, present characteristics for which data were extracted (e.g., study size, PICOS, follow-up period) and provide the citations.	Table 1
Risk of bias within studies	19	Present data on risk of bias of each study and, if available, any outcome level assessment (see item 12).	Figure 2
Results of individual studies	20	For all outcomes considered (benefits or harms), present, for each study: (a) simple summary data for each intervention group (b) effect estimates and confidence intervals, ideally with a forest plot.	Figure 3, 4, 5
Synthesis of results	21	Present results of each meta-analysis done, including confidence intervals and measures of consistency.	11, 12, 13, Figure 3
Risk of bias across studies	22	Present results of any assessment of risk of bias across studies (see Item 15).	11, 12, 13, Figure 3
Additional analysis	23	Give results of additional analyses, if done (e.g., sensitivity or subgroup analyses, meta-regression [see Item 16]).	-
**DISCUSSION**			
Summary of evidence	24	Summarize the main findings including the strength of evidence for each main outcome; consider their relevance to key groups (e.g., healthcare providers, users, and policy makers).	14
Limitations	25	Discuss limitations at study and outcome level (e.g., risk of bias), and at review-level (e.g., incomplete retrieval of identified research, reporting bias).	16
Conclusions	26	Provide a general interpretation of the results in the context of other evidence, and implications for future research.	17
**FUNDING**			
Funding	27	Describe sources of funding for the systematic review and other support (e.g., supply of data); role of funders for the systematic review.	18

For peer review only - http://bmjopen.bmj.com/site/about/guidelines.xhtml

PRISMA 2009 Checklist

From: Moher D, Liberati A, Tetzlaff J, Altman DG, The PRISMA Group (2009). Preferred Reporting Items for Systematic Reviews and Meta-Analyses: The PRISMA Statement. PLoS Med 6(6): e1000097. doi:10.1371/journal.pmed1000097

For more information, visit: **www.prisma-statement.org**.

For peer review only

For peer review only - http://bmjopen.bmj.com/site/about/guidelines.xhtml
